# Polyphenolic and Biogenic Volatile Organic Compound Profiles of Endemic Metallophytes From the Thallium‐Rich Allchar Mining Area

**DOI:** 10.1002/cbdv.71761

**Published:** 2026-09-27

**Authors:** Marinela Cvetanoska, Marina Stefova, Katerina Bačeva Andonovska, Jasmina Petreska Stanoeva

**Affiliations:** ^1^ Institute of Chemistry, Faculty of Natural Sciences and Mathematics Ss. Cyril and Methodius University in Skopje Skopje Republic of North Macedonia; ^2^ Research Centre for Environment and Materials Macedonian Academy of Sciences and Arts Skopje The Republic of North Macedonia

**Keywords:** biogenic volatile organic compounds, endemic metallophytes, flavonoids, metalliferous habitats, phenolic acids

## Abstract

The Allchar Mining Area (North Macedonia) is an extreme metalliferous environment enriched in Tl, As and Sb that supports diverse endemic metallophytes. This study investigates metabolic profiles in selected species through integrated analysis of polyphenols (HPLC/DAD/MS^n^) and biogenic volatile organic compounds (BVOCs, headspace GC–MS). A total of 78 polyphenols and 123 volatile compounds were identified. *Knautia caroli‐rechingeri* and *Centaurea leucomalla* were dominated by caffeoylquinic acid derivatives, in which reached 405.40 mg/g dry herb in *K. caroli‐rechingeri*. *Thymus thracicus* var. *alsarensis* showed a distinct profile rich in rosmarinic and salvianolic acids, whereas Tl‐hyperaccumulating *Viola* species were characterized by flavone and flavonol glycosides together with a markedly reduced diversity of phenolic acids. BVOC profiles also differed markedly among species, including terpene‐rich profiles in *Thymus*, aldehyde‐dominated compositions in *Knautia* and *Centaurea*, and non‐terpenoid‐enriched profiles in *Viola* species. Multivariate analysis confirmed species‐specific metabolic patterns and significant concordance between polyphenolic and BVOC profiles (Mantel *ρ* = 0.72 and *p* = 0.008), while no significant association with reported Tl accumulation was observed. Metabolomic and metal accumulation patterns highlight species‐specific biochemical characteristics associated with life in metalliferous habitats.

## Graphical abstract

Six plant taxa endemic to the thallium‐rich Allchar Mining Area (North Macedonia) were profiled by HPLC/DAD/MSn and headspace GC–MS. Seventy‐eight polyphenols and 123 biogenic volatile organic compounds were identified, and multivariate analysis showed congruent, species‐specific polyphenolic and volatile signatures with no significant association with previously reported thallium accumulation.

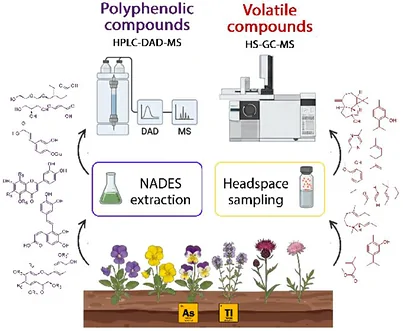

## 1 Introduction

The Allchar Mining Area, located on the slopes of Kozuf Mountain, represents one of the most geochemically distinctive environments worldwide. This ancient mining site has been exploited for centuries and is globally recognized as the only known deposit of the thallium‐bearing mineral lorandite, accompanied by elevated concentrations of arsenic, antimony, gold, nickel, lead and cadmium [1]. Owing to these unique geochemical characteristics, the locality has attracted considerable scientific interest, including its proposed relevance in solar neutrino detection and environmentally clean energy research.

Despite these extreme geochemical conditions, a notable diversity of plant species has been recorded in this area, including a significant number of endemic taxa. According to Micevski and Matevski [2], the site is of particular importance due to the presence of locally restricted endemic species such as *Odontarrhena kavadarcensis*, *Centaurea kavadariensis*, *Centaurea leucomalla*, *Knautia caroli‐rechingeri*, *Viola allchariensis*, *Viola arsenica*, and *Viola × halacsyana*. These taxa represent an important model system for studying plant survival and adaptation under extreme metalliferous conditions.

Soil contamination with heavy metals and other toxic elements is widely recognized as a major environmental issue. Elevated concentrations of potentially toxic elements such as Cd, Co, Cr, Hg, Mn, Pb, and Zn have been reported to exert pronounced phytotoxic effects, resulting in inhibition of key physiological and biochemical processes, reduced biomass, and, in severe cases, plant death [3]. Nevertheless, such general responses contrast with the persistence of metallophyte species observed in extreme environments such as the Allchar.

Certain plant species have evolved specialized mechanisms for metal and metalloid tolerance, including sequestration in metabolically active compartments or incorporation into stable chelate complexes [4, 5]. Plants adapted to metal‐rich substrates while maintaining normal growth are defined as metallophytes [6, 7]. These species are of particular importance for understanding ecological adaptation and evolutionary processes, as well as for applications in phytoremediation [8].

In metalliferous habitats, abiotic stress significantly alters plant chemical composition, often through modulation of secondary metabolism. A central mechanism involves oxidative stress induced by reactive oxygen species (ROS), both radical and non‐radical (O_2_•^−^, •OH, H_2_O_2_, ^1^O_2_) that may cause membrane damage, reduced photosynthesis, and disruption of genetic processes [9]. Metals such as Cu, Pb, Cr, and As can directly induce ROS formation, whereas Cd acts mainly through enzyme inhibition [5, 10].

To mitigate oxidative stress, plants rely on antioxidant systems composed of enzymatic and non‐enzymatic components. Among the latter, polyphenolic compounds are of particular importance due to their redox activity, metal‐chelating properties, and regulatory roles in stress signaling [5, 11]. Flavonoids, especially aglycones, are highly effective in formation of stable complexes with metal ions and reduce oxidative damage [12, 13]. Recent reviews confirm that an increased flux through the phenylpropanoid pathway, and the consequent accumulation of phenolic acids and flavonoids, is among the most consistently reported biochemical responses of plants to abiotic stress, including exposure to potentially toxic elements [14, 15].

Heavy metals exposure has been shown to activate the phenylpropanoid pathway resulting in increased synthesis of flavonoids and related compounds. Such responses have been reported in several plant species under Cu^2^^+^, Cd^2^^+^, and combined metals [16, 17]. However, these effects are highly variable and depend on both plant species and metal type. For example, in *Spinacia oleracea*, mercury exposure increased flavonoid content but reduced total phenols, while arsenic had no significant effect [18]. In *Larix sibirica*, aluminum exposure increased multiple phenolic groups, whereas in polluted field conditions *Spiraea media* showed reduced flavonoid aglycones and increased phenylcarboxylic acids compared to control sites [19, 20]. Overall, these findings indicate highly complex, species‐specific, and pollutant‐dependent antioxidant responses.

In addition to polyphenols, biogenic volatile organic compounds (BVOCs), as major constituents of essential oils, represent another important class of secondary metabolites involved in plant stress responses. However, their role under heavy metal exposure remains poorly understood and is largely based on controlled experimental studies. In *Ocimum basilicum*, low arsenic concentrations have been reported to increase essential oil production, whereas higher concentrations (>150 mg/kg) reduce it and alter volatile composition, including increased linalool and decreased eucalyptol levels [21]. Due to the limited available data, no general conclusions can yet be drawn regarding BVOC responses under metal stress. Untargeted metabolomic approaches based on liquid chromatography coupled to multistage or high‐resolution mass spectrometry, increasingly supported by molecular networking, have proven effective for resolving such complex mixtures of specialized metabolites and were adopted here for that purpose [22].

Previous work on the Allchar flora has focused almost exclusively on the uptake, accumulation and tissue distribution of Tl and As [23, 24, 25]. The specialized‐metabolite chemistry of these taxa, in contrast, is largely unknown: apart from a single report on *Thymus thracicus* var. *alsarensis* [26], no polyphenolic profile has been published for any of the Allchar endemics, and no BVOC profile has been published for any of them.

Therefore, in the present study, for the first time, a comprehensive analysis of the polyphenolic profile and BVOCs of six selected endemic metallophytes from the Allchar Mining Area is performed using HPLC/DAD/MS^n^ and headspace GC–MS, respectively. Particular emphasis is placed on characterizing their specialized‐metabolite responses under long‐term natural exposure to the metalliferous conditions of the area, characterized by elevated concentrations of Tl, As, and Sb and on evaluating differences in comparison with botanically related species from non‐metalliferous environments. By combining two complementary analytical approaches, this study provides an integrated characterization of the major specialized metabolites associated with these endemic metallophytes and provides a chemical baseline for interpreting how these species persist under the metalliferous conditions of the area.

Because the studied taxa are endemic to the Allchar area and naturally occur under its metalliferous conditions, appropriate non‐metalliferous control populations of the same taxa are not available. Therefore, comparisons with botanically closely related species occurring on non‐metalliferous soils were used as a contextual reference, while acknowledging that such comparisons cannot unequivocally distinguish the effects of metal exposure from species‐specific, phylogenetic, phenological, and other environmental differences.

## 2 Results and Discussion

### 2.1 Analysis of BVOCs

Biogenic volatile organic compounds represent important products of both primary and secondary plant metabolism. Their qualitative and quantitative composition provides information on chemotaxonomic differentiation, as well as on specific environmental and growing conditions.

**FIGURE 1:**
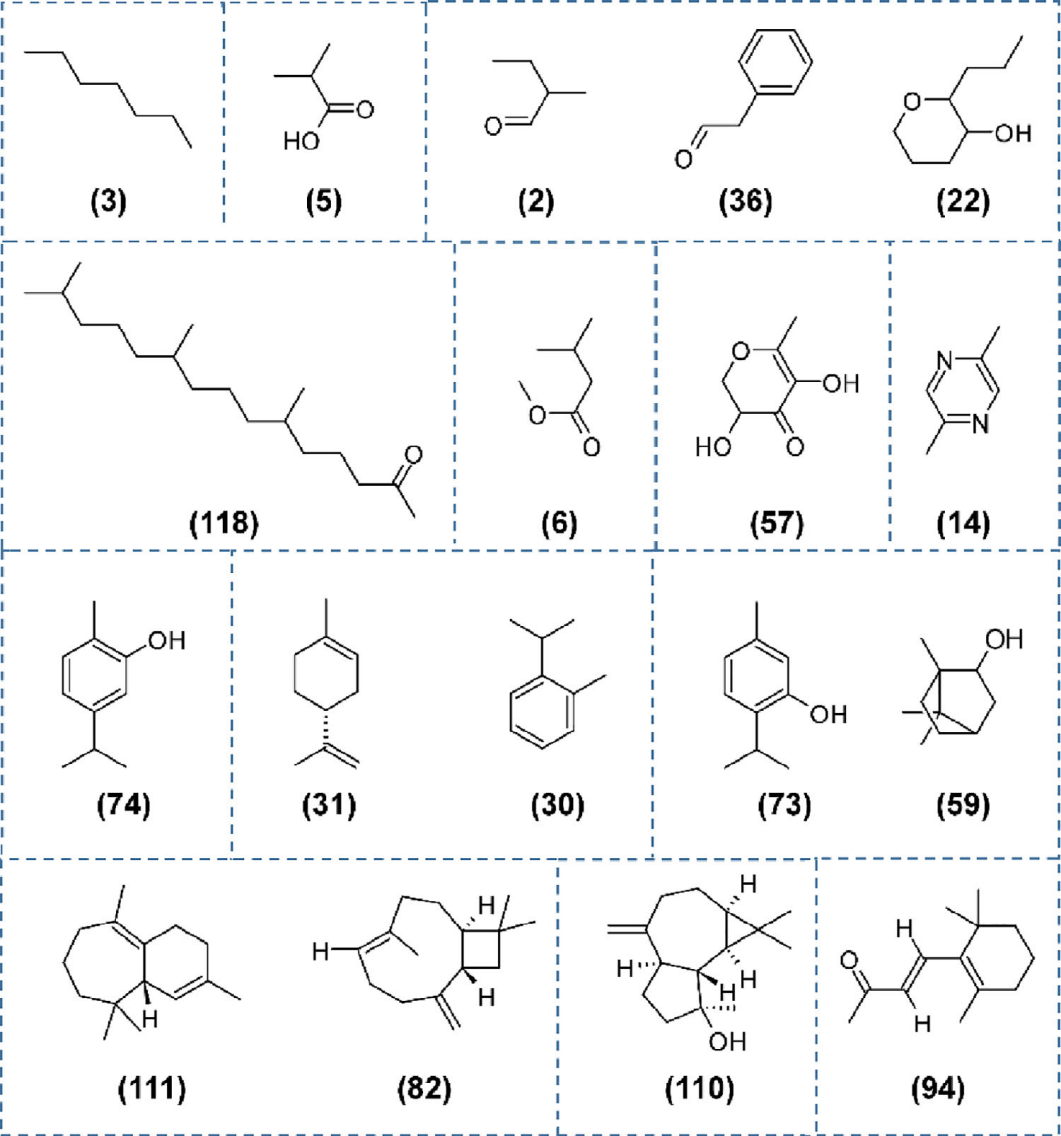
Chemical structures of detected BVOCs. The numbers in parentheses correspond to the compound numbers listed in Table S1.

**FIGURE 2:**
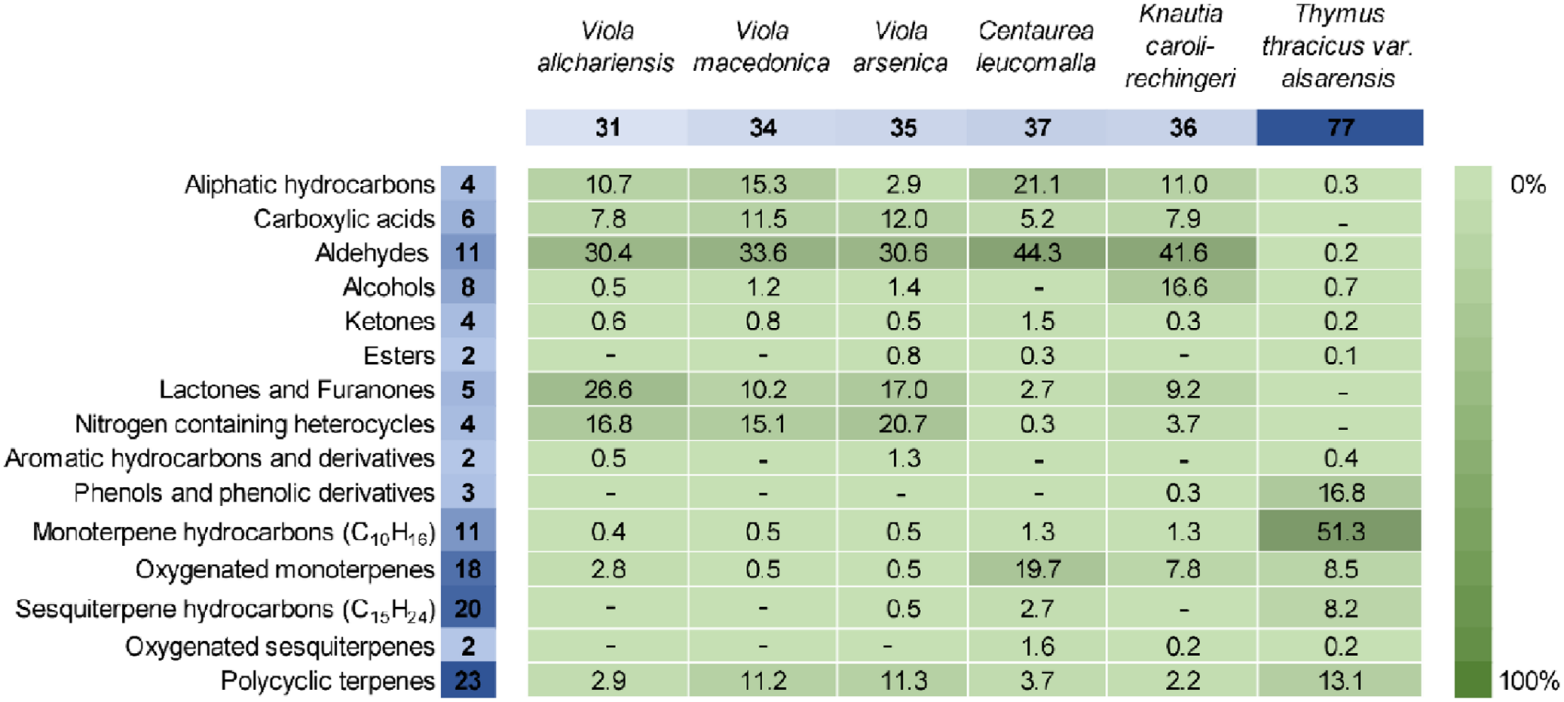
Heatmap of relative abundance (%) of BVOCs in the analyzed samples (green color) and number of different structures identified (blue color).

Qualitative analysis of volatile profiles revealed high chemical diversity among the investigated species. Representative chromatograms for all samples are provided in the (Figure S1a–f). A total of 123 compounds were identified, with terpenes representing the dominant group (74 compounds), including 23 polycyclic terpenes, 20 sesquiterpenes, 18 oxygenated monoterpenes, 11 monoterpenes, and 2 oxygenated sesquiterpenes. In addition, non‐terpenoid compounds were detected, including 11 aldehydes, 8 alcohols, 6 carboxylic acids, and smaller number of aliphatic hydrocarbons, esters, ketones, lactones, aromatic compounds, and nitrogen‐containing heterocyclic compounds. A representative from each group is shown in Figure 1, while Figure 2 presents a summary of the identified compounds; the complete detailed data are provided in Table S1. The dominance of terpenes is consistent with their well‐established contribution to the aroma and the bioactivity of plant essential oils [27].

The highest number of different BVOCs have been identified in *Thymus thracicus* var. *alsarensis* (77 compounds) with monoterpenes being the predominant class (51.3% of total BVOCs, Figure 2). The most abundant monoterpenes were D‐limonene (29.7%) and *γ*‐terpinene (7.8%), both of which were not detected in any of the other investigated species. No previous data are available for the volatile profile of *T. thracicus*, however, marked differences were observed compared to *T. vulgaris* (common thyme), in which thymol (oxygenated monoterpene) is reported as dominant compound (72%), *γ*‐terpinene occurs only in trace amounts, and D‐limonene is absent [28]. In the present study, phenolic derivatives were also prominent in *T. thracicus*, including carvacrol (2‐methyl‐5‐(1‐methylethyl)phenol) (16.2%) and carvacryl acetate (0.6%), both uniquely detected in this species. Polycyclic terpenes also had relatively high abundance (13.1%), where *β*‐ionone was not detected, which is an exception only for this analyzed sample. Of the 20 sesquiterpenes detected across all samples, 19 were present in *T. thracicus*, indicating their potential chemotaxonomic relevance. Sesquiterpenes were not detected in previous studies on the most widely distributed species *T. vulgaris* [28].

In *Centaurea leucomalla*, 37 volatile compounds were identified, with aldehydes as the dominant group (44.3%), followed by aliphatic hydrocarbons (21.1%). Benzeneacetaldehyde (10.4%) was the most abundant aldehyde and has also been reported in *C. scabiosa* [29] and *C. triumfettii* [30]. Oxygenated monoterpenes accounted for 19.7%, with thymol as the major representative (19.3%), showing a markedly higher abundance compared to other studied species. Notably, thymol has not been previously reported in other *Centaurea* species [29, 30, 31, 32]. In contrast, sesquiterpenes, which are typically dominant in the genus [30, 32], were present only in low amounts in *C. leucomalla* (1.6% and 2.7% for oxygenated and non‐oxygenated sesquiterpenes, respectively).

The *Knautia caorli‐rechingeri* sample is characterized by 36 different BVOCs. Aldehydes were again predominant (41.6%) with 2‐methylbutanal (19.4%) and benzeneacetaldehyde (10.6%) as major constituents. Alcohols represented 16.6%, a markedly higher proportion compared to all other analyzed samples, where this group remained below 1.5%. The terpene profile is represented by oxygenated monoterpenes (7.8%) and polycyclic terpenes (2.2%), but no sesquiterpenes were detected. The highest content of oxygenated monoterpenes was observed for thymol (4.2%). Chrysanthenol and (−)‐myrtenol were uniquely detected in this sample. This research represents one of the first detailed BVOC characterizations of the genus *Knautia*.

In the three *Viola* species (*V. macedonica*, *V. arsenica*, and *V. allchariensis*), aldehydes were consistently the dominant class (33.6%, 30.6%, and 30.4%, respectively). 2‐Methylbutanal was the most abundant compound in all three species, as also observed in the samples of *K. caroli‐rechingeri* and *C. leucomalla*. A notable feature of these species was the relatively high proportion of furanones, lactones, and nitrogen‐containing heterocycles (10.2%–26.6%), which was considerably higher than in the other investigated taxa. Terpenes were present only in low amounts, with the exception of polycyclic terpenes (2.9%–11.3%). In this class, methyl salicylate was identified as a characteristic compound, a component that was dominant in *V. dubyana* and *V. calcarata*, according to previous research [33]. According to published data, the most widely distributed *Viola* species in Europe is *V. odorata*, whose composition is dominated by linalool and eugenol [34], two compounds that were not detected at all in the samples of these study.

Overall, the results indicate that endemic species from the Allchar area exhibit distinct and species‐specific BVOC profiles. Although these patterns may be associated with the specific environmental conditions of the Allchar habitat, the present data do not allow the contribution of individual metals to BVOC composition and abundance to be established. A potential role of metal‐related environmental conditions therefore remains a hypothesis that requires further investigation under controlled experimental conditions.

### 2.2 Polyphenolic Profile

#### 2.2.1 Qualitative Analysis

**FIGURE 3:**
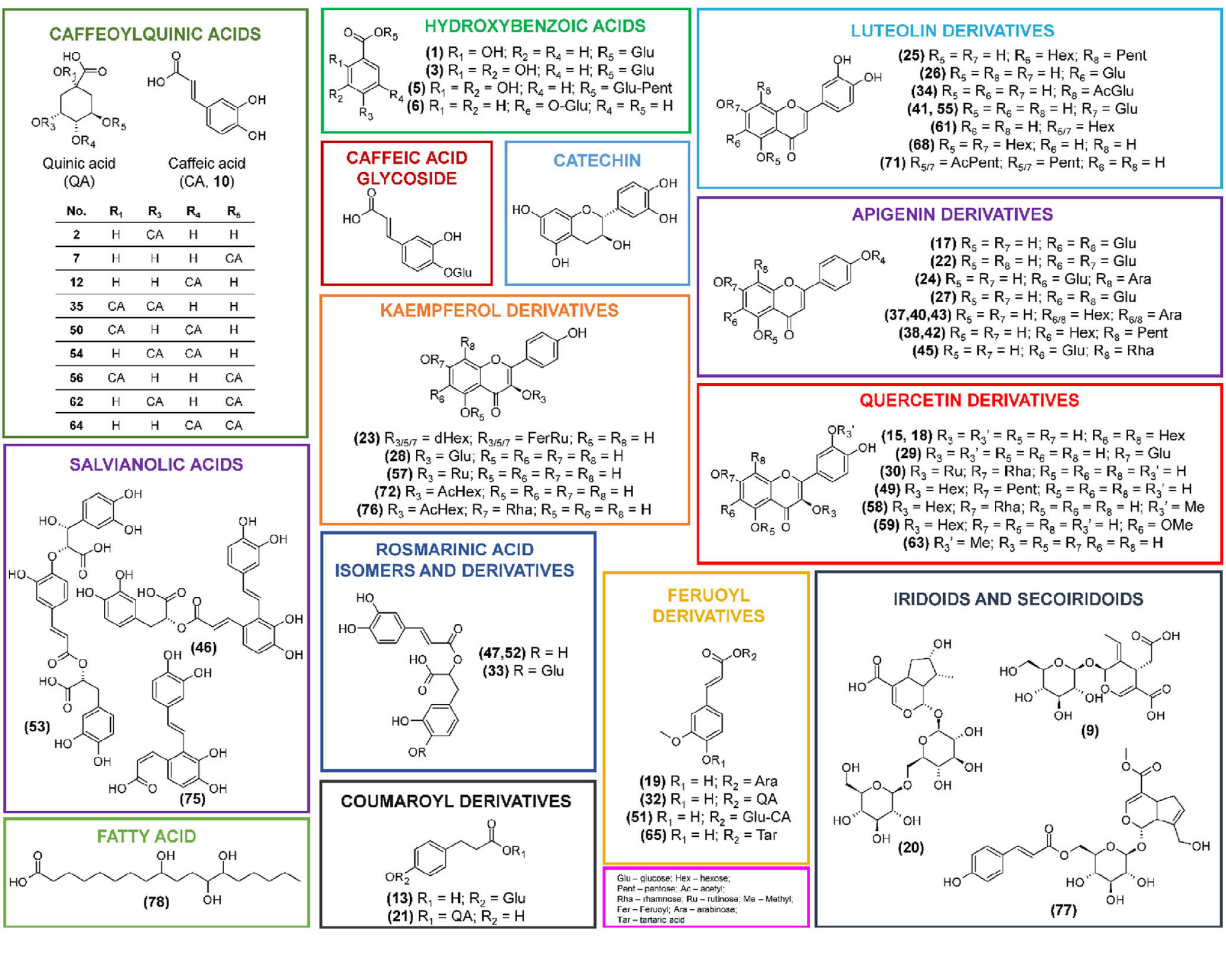
Chemical structures of identified polyphenolic compounds. The numbers in parentheses correspond to the compound numbers listed in Table 1.

A total of 78 compounds were detected and identified: phenolic acids (31), flavones (21), flavonols (20), iridoids and secoiridoids (4), flavan‐3‐ols (1) and fatty acids (1). Compound annotation was based on UV–vis spectra, MS and MS*^n^* fragmentation patterns, and comparison with literature data, following an untargeted annotation workflow analogous to that applied to other complex leaf extracts [22]. Full structural information, including retention times, UV maxima, and MS data, is provided in Table 1, while representative chemical structures are shown in Figure 3. Representative chromatograms for all samples are provided in the (Figure S2).

**TABLE 1:** Retention times (*t_R_*), UV maxima, and MS data for identified compounds.

Nos.	Compound names	*t*_R_/min	*λ*_max_	[M‐H]^−^	MS^2^	Detected in sample^a^
Phenolic acid derivatives						
Caffeoyl derivatives						
Mono‐ and dicaffeoyl derivatives						
**2**	3‐Caffeoylquinic acid	7.5	244, 300sh, and **326**	353	**191** ^b^, 179, and 135	CL and KC
**7**	5‐Caffeoylquinic acid	13.3	240, 298sh, and **326**	353	191	CL and KC
**10**	Caffeic acid	15.4	244, 294sh, and **326**	179	161, 147, and **135**	TA
**12**	4‐Caffeoylquinic acid	16.6	240, 298sh, and **326**	353	191	CL and KC
**35**	1,3‐Dicaffeoylquinic acid	22.2	244, 300sh, and **326**	515	**353**, 191, and 179	KC
**50**	1,4‐Dicaffeoylquinic acid	25.1	244, 298sh, and **328**	515	**353**, 317, 299, 255, and 203	KC
**54**	3,4‐Dicaffeoylquinic acid	26.7	242, 296sh, and **330**	515	**353** and 299	CL
**56**	1,5‐Dicaffeoylquinic acid	27.5	242, 294sh, and **332**	515	**353** and 331	CL
**62**	3,5‐Dicaffeoylquinic acid	29.9	236, 296sh, and **326**	515	**353**, 191, 179, and 173	KC
**64**	4,5‐Dicaffeoylquinic acid	30.4	248, 296sh, and **326**	515	**353** and 299	CL
	Salvianolic acids					
**46**	Salvianolic acid A	24.5	234, 254, 288, **310**, and 344sh	493	295	TA
**53**	Salvianolic acid K	26.6	236, 288sh, and **324**	555	**493** and 359	TA
**73**	Isosalvianolic acid A	34.3	234, 288, and **332**	493	359, **331**, 197, 179, and 161	TA
**75**	Salvianolic acid F	36.8	232, 272, and **310**	313	269, 243, and **161**	TA
	Glycosides					
**4**	Caffeic acid 3‐*O*‐glucoside	9.6	252, 290, and **322**	341	297, **179**, and 135	KC
	Depsides (caffeic acid esters)					
**33**	Rosmarinic acid glucoside	21.3	256 and **328**	521	**359** and 161	TA
**47**	Rosmarinic acid isomer 1	24.9	234, 284, and **316**	359	223, 197, 179, and **161**	TA
**52**	Rosmarinic acid isomer 2	25.9	236, 288sh, and **330**	359	223, 197, 179, **161**, and 133	TA
	Coumaroyl derivatives					
**13**	Coumaroyl pentoside	16.9	232 and **314**	295	163	KC
**21**	Coumaroylquinic acid	19.3	314	337	**191** and 173	CL and KC
	Feruloyl derivatives					
**19**	Feruloyl arabinoside	18.3	244, 296sh, and **328**	325	193	KC
**32**	5‐Feruloylquinic acid	20.9	236, 294sh, and **326**	367	191	CL
**51**	Caffeoyl feruloylquinic acid	29.9	244, 294sh, and **328**	529	367, **353**, and 191	KC
**65**	Feruloyl tartaric acid (fertaric acid)	30.7	236, 298sh, and **326**	325	**193**, 178, 149, and 135	KC
	Hydroxybenzoic acid derivatives					
**1**	Gentisoyl glucoside	6.7	316	315	**153** and 108	CL
**3**	Protocatechuic acid glucoside	7.8	238 and **316**	315	156	VAr
**5**	Protocatechuic acid glucoside pentoside	10.2	234 and **312**	447	**315**, 271, 207, and 152	VAr
**6**	Hydroxybenzoic acid glucoside	13.1	**298** and 326	299	137	VAr, VAl, and VM
	Other					
**8**	Sinapic acid hexoside	14.1	238, 294sh, and **312**	369	**223**, 205, and 129	VAr
**14**	Dimer of 3‐(3,4‐dihydroxyphenyl) lactic acid	16.9	278	377	**359** and 161	TA
**16**	Hydroxyjasmonic acid‐*O*‐hexoside	17.9	272	387	369, **207**, and 163	TA
	Flavones					
	Apigenin derivatives					
**17**	Vicenin‐2 (apigenin‐6,8‐di‐*C*‐glucoside)	18, 0	236, **272**, and 338	593	575, 503, **473**, 383, and 353	VAr, VAl, VM, and CL
**22**	Apigenin 6‐*C*‐glucosyl‐7‐*O*‐glucoside	19.3	236, 270, and **336**	593	473, 431, 341, **311**, and 283	KC
**24**	Apigenin 6‐*C*‐glucoside 8‐*C*‐arabinoside (schaftoside)	19.6	**272** and 326	563	545, 519, 503, **473**, 443, 383, and 353	VAl and VM
**27**	Apigenin‐di‐*C*‐glucoside	20.5	234, 272, and **334**	593	503, **473**, and 353	CL
**37**	Apigenin‐*C*‐rhamnoside‐*C*‐hexoside isomer	22.3	272 and **334**	577	559, 503, **473**, 383, and 353	VAr, VAl, and VM
**38**	Apigenin 8‐*C*‐pentoside 6‐*C*‐hexoside	22.6	232, 272, and **336**	563	545, 503, **473**, 443, 382, and 353	CL
**40**	Apigenin‐*C*‐rhamnoside‐*C*‐hexoside isomer	23.0	258, 272, and **336**	577	559, **503**, 473, 457, 383, and 353	VAr
**42**	Apigenin 8‐*C*‐hexoside 6‐*C*‐pentoside	23.3	234, 270, and **334**	563	545, 503, **473**, 443, 382, and 353	CL
**43**	Apigenin‐*C*‐rhamnoside‐*C*‐hexoside isomer	23.4	236, 272, and **336**	577	**473**, 383, and 353	VAr and VM
**44**	Acacetin‐*C*‐glucoside	23.8	234, 270, and **336**	445	**325**, 297, 282, and 231	KC
**45**	Apigenin‐6‐*C*‐glucoside‐8‐*C*‐rhamnoside	24.1	236, 270, and **340**	577	559, 487, **457**, 413, 383, and 353	VAr, VAl, and VM
	Luteolin derivatives					
**25**	Luteolin 6‐*C*‐hexoside‐8‐*C*‐pentoside	20.0	274 and **330**	579	**489**, 459, 399, and 369	VAr
**26**	Isoorientin (luteolin‐6‐*C*‐glucoside)	20.4	238, 258, 268, and **350**	447	429, 357, and **327**	KC
**31**	Chrysoeriol‐*C*‐glucoside	20.9	244, 258, 268, and **350**	461	**341**, 313, and 299	KC
**34**	Luteolin‐8‐*C*‐(6”acetyl)‐*β*‐D‐glucopyranoside	21.6	272 and **350**	489	**447** and 327	VM
**41**	Luteolin‐7‐*O*‐glucoside	23.3	236, 284, and **330**	447	285	TA
**55**	Luteolin‐4’‐*O*‐glucoside	27.4	236, 266, 298, and **334**	447	327, **285**, and 255	KC
**61**	Luteolin *O*‐hexoside	29.8	232, 286, and **332**	447	285	TA
**68**	Luteolin *O*‐dihexoside	31.3	232, 284, and **332**	609	447 and **285**	ТА
**71**	Luteolin *O*‐acetyl‐dipentoside	32.4	230, 266, and **346**	591	549, **531**, and 285	ТА
	Isoflavone					
**39**	Genistein‐*C*‐rhamnoside‐*C*‐hexoside	22.7	**266** and 350	577	503, **473**, 457, 413, 383, and 353	VAl
	Flavonols					
	Quercetin derivatives					
**15**	Quercetin di‐*C*‐hexoside	16.9	262, 290, and **356**	609	619, **489**, 399, and 369	CL
**18**	Quercetin di‐*C*‐hexoside	18.1	234, 252, 272, and **348**	609	619, **489**, 399, and 369	CL
**29**	Quercetin 7‐*O*‐glucoside	20.7	236, 254, 282, and **346**	463	301	TA
**30**	Quercetin‐rhamnosyl‐rutinoside	20.7	256 and **354**	755	609, 591, 489, **301**, and 271	VAl
**48**	Isorhamnetin‐rhamnosyl‐rutinoside	24.9	256 and **354**	769	**623** and 315	VAr
**49**	Quercetin‐3‐*О*‐hexoside‐7‐*О*‐pentoside	24.9	276 and **344**	595	301	CL
**58**	Isorhamnetin 3‐*O*‐hexoside 7‐*O*‐rhamnoside	28.5	254 and **354**	623	**315** and 300	VAr, VAl, and VM
**59**	Patuletin 3‐*O*‐hexoside	28.7	246, 292, 316, and **348**	493	331	CL
**63**	Isorhamnetin (3‐*O*‐methylquercetin)	30.2	286 and **336**	315	300	TA
**67**	Quercetin‐*O*‐acetylhexoside	31.1	274 and **346**	505	301	CL
**70**	Patuletin acetyl hexoside	31.8	240, 258, and **354**	535	331	CL
	Kaempferol derivatives					
**23**	Kampferol‐*O*‐deoxyhexoside‐*O*‐feruloylrutinoside	19.4	258, 266, and **350**	915	**769**, 593, 431, and 285	VAr
**28**	Kaempferol 3‐*O*‐glucoside	20.5	256, 268, 292, and **350**	447	423, 357, **327**, and 285	VAr and VM
**57**	Kaempferol‐3‐*O*‐rutinoside	27.9	238, 266, 304, and **352**	593	285	VAr, VAl, and VM
**60**	Kaempferol dipentoside	28.9	254 and **350**	549	417, 327, and **285**	TA
**66**	Kaempferol acetyl pentosyl‐hexoside	30.7	232, 254, and **352**	621	**579**, 561, and 285	TA
**72**	Kaempferol‐*O*‐acetylhexoside	33.0	234, 266, and **342**	489	285	CL
**76**	Kaempferol 3‐(6''‐acetylglucoside)‐7‐rhamnoside	38.2	236, 268, and **314**	635	575, 489, 349, and **285**	KC
	Other					
**36**	Myricetin‐hexoside	22.2	238, 260, and **358**	479	317	CL
**74**	Flavanomarein	34.7	250, 298sh, and **330**	449	**287** and 173	KC
	Flavan‐3‐ol					
**11**	Catechin	15.9	280	289	271 and 229	VM and VAr
	Iridoids and secoiridoids					
**9**	Oleoside (secologanoside)	15.4	240, 296sh, and **324**	389	**345**, 209, 165, and 121	KC
**20**	Loganic acid glucoside	18.9	266	537	**375** and 179	VM and VAl
**69**	Unknown 1	31.5	292	429	385, **249**, 205, 179, and 161	VM, VAl, and VAr
**77**	*p*‐Coumaroylgeniposidic acid	41.8	260 and **316**	519	477, 459, **163**, and 145	TA
	Fatty acids					
**78**	Trihydroxy‐octadecenoic acid	46.7	228, **276**, and 322	329	311, 293, **229**, 211, and 183	TA

^a^ CL—*C. leucomalla*, KC—K. *caroli‐rechingeri*, TA—*T. thracicus* var. *alsarensis*, VAl—*V. allcharinesis*, VAr—*V. arsenica*, VM—*V. macedonica*.

^b^ Numbers in bold refer to base fragment ion in MS^2^.

*Phenolic acids* (31 compounds) constituted the largest group, showing characteristic UV maxima at 240–250 nm and 320–330 nm, consistent with hydroxycinnamate‐type structures. Among them, caffeic acid derivatives (19 compounds) were dominant, including mono‐ and dicaffeoylquinic acids, salvianolic acids, glycosides, and depsides (caffeic acid esters). In general, *Viola* species exhibited lower diversity of phenolic acid derivatives compared to *Knautia*, *Thymus*, and *Centaurea*, in agreement with previous reports [35, 36]. Caffeic acid (**10**) was detected only in *T. thracicus*, whereas monocaffeoylquinic acids (**2**, **7**, and **12**) were present in both *Centaurea* and *Knautia*. In a previous study of this species, caffeic acid was detected only in glucoside form [26].

All six isomers of dicaffeoylquinic acids (**35**, **50**, **54**, **56**, **62**, and **64**) were identified in these genera, showing characteristic MS fragmentation (*m/z* 515 → 353). All of the structures classified as salvianolic acids and depsides were detected in the *T. thracicus* sample, of which only compound **46** was previously reported [26].

Rosmarinic acid derivatives (**33**, **47**, and **52**) were mainly found in *T. thracicus*, consistent with a pronounced contribution of esterified caffeic acid conjugates to the phenolic pool of this taxon. Compound **14** was also detected in *Thymus*, supporting the diversity of caffeic‐acid‐derived structures in this genus previously reported in three other *Thymus* species from Croatia [37]. In contrast, coumaric acid derivatives (**13** and **21**) and feruloyl derivatives (**19**, **32**, **51**, and **65**) were restricted to *K. caroli‐rechingeri* and *C. leucomalla*, suggesting species‐specific allocation of hydroxycinnamate metabolism. Sinapic acid hexoside (**8**) was detected exclusively in *V. arsenica*, while hydroxyjasmonic acid‐*O*‐hexoside (**16**) was present in *Thymus* species, in agreement with earlier reports (for *T. thracicus*) [26, 38]. Hydroxybenzoic acid derivatives (**1**, **3**, **5**, and **6**) were characteristic for *Viola* species. Although, hydroxybenzoic acids, such as gentisic and protocatechuic acids have been reported in several *Viola* species [39, 40], but reports of their glycosylated derivatives remain scarce.

*Flavones* (21 compounds) included derivatives of apigenin, luteolin, chrysoeriol, and acacetin occurring mainly as *C*‐ and *O*‐glycosides. Additionally, C‐glycosylated derivative of genistein was detected, as the only representative of *isoflavones*. C‐Glycosylated apigenin derivatives were widely distributed across most species, reflecting a conserved biosynthetic pattern, whereas *T. thracicus* lacked apigenin derivatives but contained luteolin *O*‐glycosides, indicating a shift in flavonoid biosynthetic routing. Luteolin and chrysoeriol as aglycones were also detected. Previous profiling studies have demonstrated that Viola, Thymus, Centaurea, and, to a lesser extent, Knautia species are rich sources of flavone derivatives, particularly apigenin‐ and luteolin‐based glycosides [41, 42]. According to literature data, among the investigated taxa, *Viola* spp. showed the strongest evidence for the occurrence of apigenin, luteolin and chrysoeriol derivatives [39], including both glycosylated forms and corresponding aglycones, whereas reports of genistein derivatives remain unclear.

*Flavonols* (20 compounds) were dominated by quercetin and kaempferol derivatives. Quercetin glycosides (**29**, **48**, **49**, **58**, **63**, **67**, and **70**) were identified via the diagnostic ion at *m/z* 301, with characteristic neutral losses of hexose, pentose, and acetylhexose moieties. Kaempferol derivatives (**23**, **57**, and **76**) were exclusively *O*‐glycosides, yielding the aglycone ion at *m/z* 285. Notably, compound **76**, identified as kaempferol 3‐(6''‐acetylglucoside)‐7‐rhamnoside, which to the best of our knowledge has not previously been reported in the genus *Knautia* and within Caprifoliaceae, indicating rare chemotaxonomic specialization under extreme environmental conditions. The compound has only been described once in the literature, in *Delphinium formosum* [43], indicating a very limited distribution. Its detection in this study therefore represents the first report for both the genus and the family, expanding the known chemotaxonomic occurrence of acetylated kaempferol glycosides.

Additionally, compound **74** was identified as flavanomarein. This compound is characteristic for the Asteraceae [44, 45] family, but it has not been reported for the *Knautia* genus, or within the family Caprifoliaceae.

*Flavan‐3‐ols* were represented by catechin (**11**), detected only in *V. macedonica* and *V. arsenica*, consistent with its known occurrence in the genus *Viola*. These compounds have been reported in *V. odorata* [46] and *V. calcarata* [47].

*Iridoids and secoiridoids* (**9**, **20**, and **77**) included oleoside derivatives, loganic acid, and coumaric acid conjugates, which were identified based on characteristic neutral losses (CO_2_, hexose) and diagnostic fragment ions. Iridoids and secoiridoids are considerably less common than flavonoids in the Viola, Thymus, Knautia, and Centaurea genera. Their occurrence appears to be strongly genus‐ and species‐dependent. Among the investigated genera, Knautia species (Caprifoliaceae) are the most likely source of iridoid‐type metabolites, as iridoids are characteristic chemotaxonomic markers of the Caprifoliaceae group [48].

A single fatty acid, trihydroxyoctadecanoic acid (**78**), was identified based on typical oxylipin fragmentation and hydroxylation patterns [49].

#### 2.2.2 Quantitative Analysis

**FIGURE 4:**
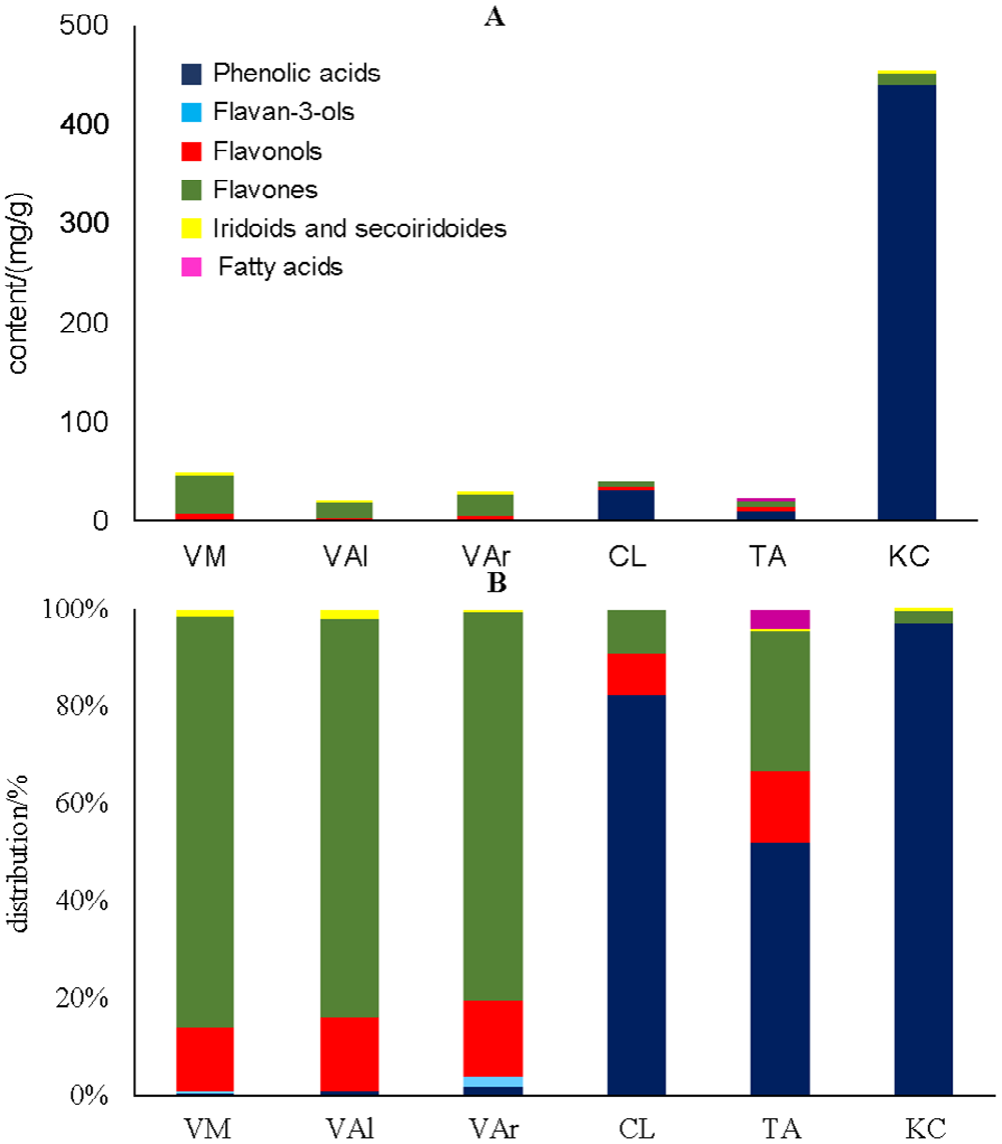
Total content of detected compounds expressed as mg/g dry herb (A) and percentage distribution (B), of all samples analyzed in triplicates.

The total content of the detected compounds varied considerably among the analyzed samples, following the order: *K. caroli‐rechingeri* (453.23 mg/g), *V. macedonica* (47.28 mg/g), *C. leucomalla* (38.12 mg/g), *V. arsenica* (28.23 mg/g), *T. thracicus* (20.86 mg/g) and *V. allchariensis* (18.04 mg/g), presented in Figure 4. The detailed distribution and content of individual compounds is provided in Table S2.

Pronounced qualitative and quantitative differences were observed in the phenolic acid derivatives. Monomeric caffeic acid derivatives, including mono‐ and dicaffeoylquinic acids, were only detected in *K. caroli‐rechingeri* and *C. leucomalla*, where they constituted the dominant portion of the total phenolic content. This agrees with previously reported dominance of caffeoylquinic acid derivatives in related *Knautia* and *Centaurea* taxa [45, 50].

The most abundant compound across all samples was 1,3‐dicaffeoylquinic acid, reaching 405.40 mg/g in *K. caroli‐rechingeri*, and also being present, though at lower levels, in the other studied species. To our best knowledge, this compound is common in artichoke and other spices [51], but it has not been reported in *Knautia* species and certainly not in such big amount. The second most abundant compound in *K. caroli‐rechingeri* was 5‐caffeoylquinic acid (28.93 mg/g), which was also detected in *C. leucomalla* (17.99 mg/g), and is considered a key biosynthetic precursor of dicaffeoylquinic acids [52].

The phytochemical profile of *C. leucomalla* was similar to that of *K. caroli‐rechingeri*, differing by the presence of 3,4‐dicaffeoyilquinic (4.81 mg/g) and 1,5‐dicaffeoylquinic acid (4.50 mg/g). The selective enrichment of dicaffeoylquinic acid derivatives is consistent with, but does not demonstrate, a contribution to antioxidative defence; a role in metal tolerance remains a hypothesis to be tested. Such enrichment has been reported by Moglia et al. [52], whose study reports elevated content of dicaffeoylquinic acids as a response to abiotic stress. It should be noted that both *K. caroli‐rechingeri* and *C. leucomalla* have been previously investigated for their capacity to accumulate toxic metalloids. It has been shown that these taxa tend to behave as excluders, retaining metals primarily in roots, as opposed to aerial parts [25, 53]. In contrast, caffeic acid was detected only in *T. thracicus*, with a low concentration of 0.16 mg/g.

In the same species, all salvianolic acids (**46**, **53**, **73**, and **75**) and derivatives of rosmarinic acid (**33**, **47**, and **52**) were observed only in *T. thracicus* var. *alsarensis*. Rosmarinic acid represented more than 50% of the total phenolic acids (5.76 mg/g), which is in agreement with previously published data for *T. vulgaris*, *T. serpyllum*, *T. trautvetteri* and other *Thymus* species [54]. Salvianolic acid K (2.06 mg/g) and isosalvianolic acid A (2.00 mg/g) were the most abundant derivatives, representing almost 40% of all phenolic acids present. High content of salvianolic acids was previously reported for *T. thracicus* [26], as well as other closely related species [54]. In previous research, the *T. thracicus* var. *alsarensis* has exhibited a differentiated metal uptake strategy where Tl is translocated to above‐ground parts of the plant, whereas As is predominantly retained in the roots [55]. Taken together, the metal accumulation pattern and the enrichment in derivatives of salvianolic and rosmarinic acid appear to be functionally linked. The selective production of these highly active phenolics likely represents and adaptive biochemical strategy that complements metal uptake mechanisms, facilitating both detoxification and tolerance.

However, differences in total abundance were not the most distinctive feature across the samples. Instead, pronounced variation was observed in the qualitative composition and relative distribution of individual constituents.

In *Viola* species, flavones constituted the dominant phenolic class, particularly apigenin derivatives. The most abundant compound was apigenin‐6‐*C*‐glucoside‐8‐*C*‐rhamnoside, with concentrations of 25.62 mg/g (*V. macedonica*), 12.66 mg/g (*V. allchariensis*), and 15.74 mg/g (*V. arsenica*). All three species also contained vicenin‐2 (0.48–2.16 mg/g), previously reported in *V. yedoensis* [39].

Flavonols represented the second most abundant group, with total concentrations ranging from 0.19 to 6.16 mg/g across all *Viola* species. The most abundant compound was kaempferol‐3‐*O*‐glucoside (5.58 mg/g in *V. macedonica*). Kaempferol‐3‐*O*‐rutinoside (0.11–0.60 mg/g), previously reported in *V. thianscianica* [56] and *V. odorata* [57], was also detected in all analyzed samples.

The predominance of flavonoid derivatives, combined with the markedly reduced presence of phenolic acids, indicates a markedly different allocation within phenolic metabolism in the *Viola* species studied here. Notably, derivatives of coumaric and ferulic acids were not detected in any of the analyzed *Viola* samples, indicating possible suppression or redirection of hydroxycinnamate biosynthesis toward flavonoid formation, a pattern repeatedly described for plants exposed to abiotic stress [14, 15].

This observation is particularly notable given that these violets are known Tl and As hyperaccumulators, reaching concentrations of up to 58 900 and 381 mg/kg, respectively [24, 58]. In contrast to typical phytochemical profiles reported for species such as *V. tricolor* and *V. odorata*, where both flavonoids and phenolic acids (including coumaric and ferulic acids) contribute significantly to the phenolic pool [39], derivatives of these hydroxycinnamic acids were not detected in any of the analyzed samples. This absence suggests a possible redirection of hydroxycinnamate biosynthesis toward flavonoid formation or a stress‐induced suppression of phenolic acid accumulation.

This diversity of uptake and translocation mechanisms reflects species‐specific physiological controls over metal transport, sequestration, and detoxification. More broadly, hyperaccumulation is recognized as a specialized adaptation enabling plants to tolerate and sequester exceptionally high concentrations of toxic elements in their tissues without detrimental effects [6, 7]. The observed differences between analyzed taxa therefore align with the concept that even closely related or co‐occurring species can employ fundamentally different strategies for coping with metalliferous soils. These findings reinforce the importance of species‐specific investigations when interpreting both metal accumulation and associated metabolic profiles.

### 2.3 Multivariate Analysis of the Metabolite Profiles and Their Relation to Previously Reported Metal Accumulation Patterns

The variability observed in both polyphenolic and BVOC profiles can be interpreted in relation to the specific geochemical conditions of the Allchar locality, characterized by elevated concentrations of Tl, As, Sb, as well as Ni, Pb, and Cd [1]. Previously reported metal accumulation patterns provide a useful framework for interpreting the metabolic differentiation observed among the studied taxa.

*Viola* species, recognized as Tl hyperaccumulators with comparatively lower As uptake [23, 24], exhibit profiles dominated by flavonoids, and characterized by a lower diversity of phenolic acids. Such a profile suggests a preferential allocation toward flavonoid biosynthesis, potentially linked to their role in intracellular antioxidant protection and metal chelation. The absence of coumaric and ferulic acid derivatives may further indicate a possible redirection of hydroxycinnamate metabolism [59].

In contrast, *Knautia caroli‐rechingeri*, reported to accumulate higher levels of As, particularly in roots [25], is characterized by a strong enrichment in caffeoylquinic acid derivatives. Given their well‐documented antioxidant properties, these compounds may contribute to mitigating oxidative stress associated with arsenic exposure. A comparable, though less pronounced, pattern is observed in *Centaurea leucomalla*, suggesting that hydroxycinnamate derivatives may be a shared feature of these two taxa; a common adaptive significance is not demonstrated by the present data.

In *Thymus thracicus* var. *alsarensis*, rosmarinic and salvianolic acid derivatives predominated, consistent with its differentiated metal partitioning, with Tl mainly accumulated in aerial parts and As retained in roots [55]. These patterns suggest that the studied taxa employ distinct phenolic metabolic strategies that may contribute to protection against metal‐associated oxidative stress. The BVOC profiles showed similarly pronounced species‐specific differentiation, with terpene‐dominated profiles in *Thymus*, aldehyde‐rich profiles in *Centaurea* and *Knautia*, and a greater contribution of non‐terpenoid compounds in *Viola*.

**FIGURE 5:**
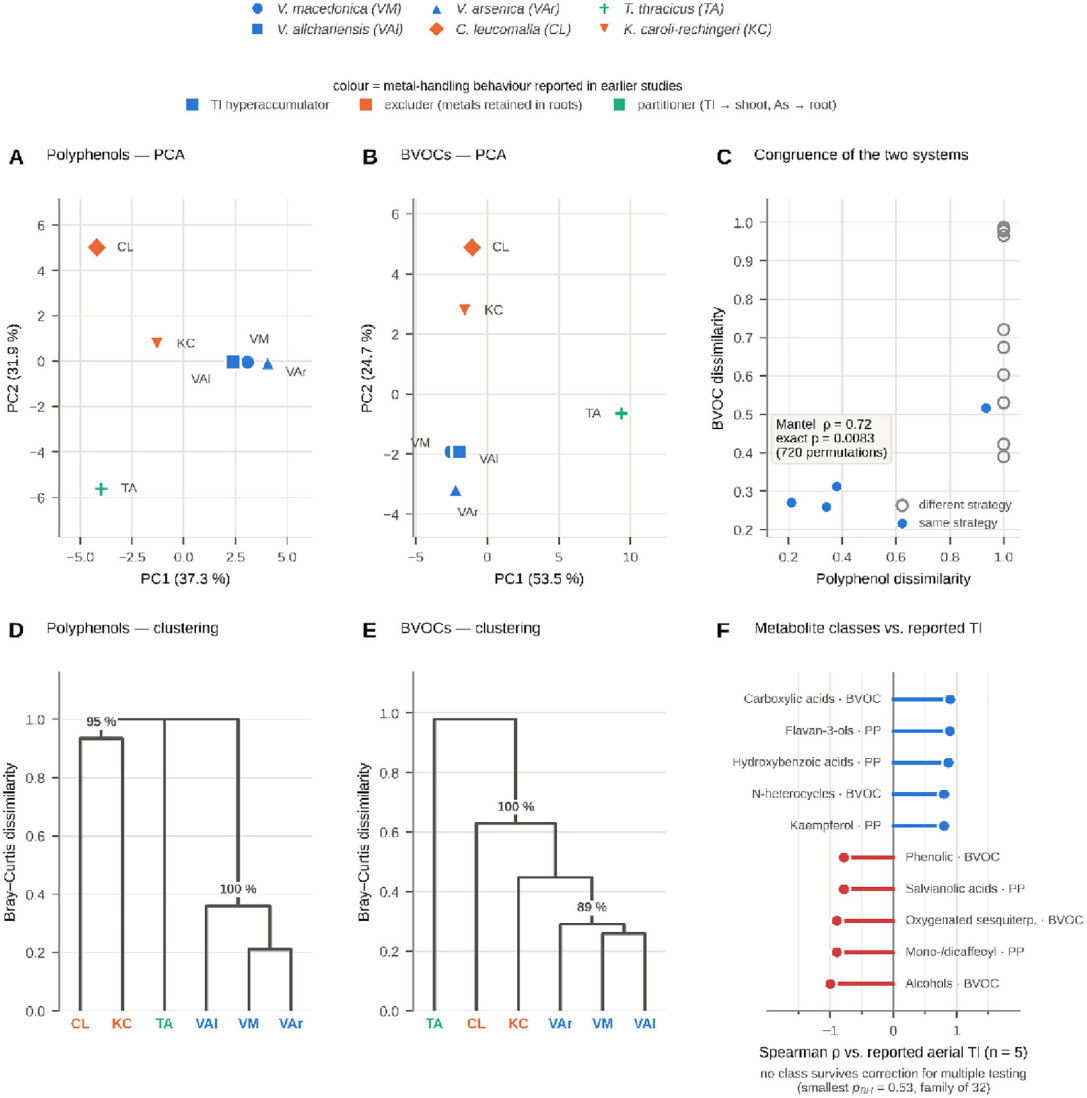
Multivariate analysis of polyphenolic and BVOC profiles of the six Allchar endemic taxa and their relationship to previously reported metal accumulation. (A, B) Principal component analysis (PCA) of polyphenolic (A) and BVOC (B) profiles. (C) Mantel test showing the relationship between polyphenolic and BVOC dissimilarities. (D, E) Hierarchical clustering of polyphenolic (D) and BVOC (E) profiles; numbers indicate bootstrap support from 2000 resamplings. (F) Spearman correlations between metabolite classes and previously reported aerial‐part Tl concentrations. PP, polyphenolic class; BVOC, volatile class. Symbols and colors indicate taxa and previously documented metal‐handling behavior, respectively; sample codes are as listed in Table 2. Summary of all tests is given in Table S6

Multivariate analysis confirmed that the observed differences represented consistent, species‐specific patterns (Figure 5). In the polyphenolic dataset, PC1 and PC2 accounted for 37.3% and 31.9% of the variance, respectively, with the three *Viola* species forming a distinct group associated mainly with apigenin *C*‐glycosides, while *T. thracicus* var. *alsarensis* and *C. leucomalla* were separated along PC2 by rosmarinic/salvianolic acid derivatives and 5‐caffeoylquinic acid, respectively (Figure 5A). For the BVOCs, PC1 explained 53.5% of the variance and separated *T. thracicus* var. *alsarensis* from the remaining taxa, primarily along a gradient involving D‐limonene, carvacrol and γ‐terpinene versus 2‐methylbutanal, acetic acid and benzeneacetaldehyde (Figure 5B). Hierarchical clustering supported these patterns, with the *Viola* group recovered in 100% and 89% of bootstrap replicates for the polyphenolic and BVOC datasets, respectively. The association of C. leucomalla and K. caroli‐rechingeri was supported in 95% of the polyphenolic replicates, while *T. thracicus* var. *alsarensis* was separated from the other taxa in 100% of the BVOC replicates (Figure 5D,E).

Importantly, the two chemically distinct metabolite datasets showed a high degree of congruence. Their dissimilarity matrices were strongly correlated (Mantel *ρ* = 0.72 and exact one‐sided *p* = 0.0083; Figure 5C), indicating that polyphenolic and volatile profiles independently captured a similar species‐specific organization. This concordance provides stronger evidence for taxon‐associated metabolic differentiation than would be obtained from either analytical platform alone.

In contrast, grouping taxa according to their previously documented metal‐handling behavior did not provide statistically significant separation. PERMANOVA comparing the three Tl‐hyperaccumulating *Viola* species with the three non‐hyperaccumulating taxa gave *F* = 3.67 for polyphenols and *F* = 2.06 for BVOCs, with an exact *p* = 0.100 in both datasets. Because only six taxa were available, the statistical resolution of this comparison was intrinsically limited; therefore, these results should not be interpreted as evidence either for or against an effect of Tl hyperaccumulation on overall metabolite composition.

A direct assessment of metabolite–metal relationships was likewise constrained by the absence of metal measurements in the present samples. Published aerial‐part concentrations were available for Tl in five taxa, As in three and Sb in only one, and originated from different studies, collection periods and, in some cases, plant organs (Table S3). Consequently, only exploratory Spearman correlations with reported aerial Tl concentrations could be evaluated. For this purpose, compound‐class composition of the six samples, expressed as the percentage of the total content of identified compounds is provided in Table S4. None of the tested metabolite classes remained significantly associated with Tl after correction for multiple comparisons (Figure 5F and Table S5). The only nominally significant association was observed for volatile alcohols (*ρ* = −1.00 and *p* = 0.017), but this value represents the minimum attainable *p*‐value for a perfect rank correlation with *n* = 5 and was not significant after Benjamini–Hochberg correction (adjusted *p* = 0.53). Similarly, total polyphenol content (*ρ* = −0.20 and *p* = 0.78) and the number of detected volatile compounds (*ρ* = −0.50 and *p* = 0.45) were not related to the reported Tl concentrations. These findings are consistent with our previous study of *T. thracicus* var. *alsarensis* [26], in which total polyphenol content was not associated with As or Tl concentrations measured in the same plants. Overall, the available evidence supports pronounced species‐specific metabolic differentiation, but does not provide sufficient evidence to attribute this variation directly to metal accumulation.

## 3 Conclusions

The present study demonstrates that endemic metallophytes from the Allchar Mining Area exhibit marked differences in both polyphenolic and BVOC composition, as revealed by complementary HPLC/DAD/MS*^n^* and headspace GC–MS analyses. Distinct metabolite patterns among the studied taxa highlight the biochemical diversity of plants inhabiting this geochemically extreme environment. These differences were reflected in the relative abundance and diversity of phenolic compounds and volatile metabolites, providing a broader biochemical perspective on the specialized metabolism of the studied endemic metallophytes.

For the first time, BVOC profiles were characterized for all six selected Allchar endemics, while polyphenolic profiles were reported for five of the six taxa. The work also represents one of the first detailed characterizations of volatile compounds within the genus *Knautia*. Several metabolites were newly reported for the corresponding taxa or taxonomic groups, including kaempferol 3‐(6′′‐acetylglucoside)‐7‐rhamnoside and flavanomarein in *K. caroli‐rechingeri*, thymol in *C. leucomalla*, and a high content of 1,3‐dicaffeoylquinic acid in *K. caroli‐rechingeri*. These findings extend the current knowledge of specialized‐metabolite diversity in the Allchar endemic flora and establish a useful chemical baseline for these poorly characterized taxa.

Multivariate statistical analysis confirmed clear species‐specific patterns in both polyphenolic and BVOC profiles. The significant concordance between the two metabolite datasets (Mantel *ρ* = 0.72 and *p* = 0.008) supports a consistent taxon‐specific organization across the two chemically distinct metabolite groups. In contrast, no significant associations were detected between metabolite classes and previously reported aerial‐part Tl concentrations after correction for multiple testing. Therefore, the observed metabolic differences cannot be directly attributed to metal accumulation based on the available data.

The observed metabolite signatures may provide hypotheses regarding potential roles of specialized metabolites in plant responses to metalliferous conditions and may contribute to understanding how these species persist in the Allchar environment. However, the present findings do not establish direct mechanisms of metal tolerance or adaptation. Further studies incorporating replicated sampling, concurrent determination of soil and plant Tl, As and Sb concentrations, comparisons of metalliferous and non‐metalliferous populations, and controlled exposure experiments are needed to clarify the functional relationship between metal exposure and species‐specific metabolic responses.

## 4 Experimental Section

### 4.1 Materials

Aerial parts of six endemic taxa were collected near the former As‐Sb‐Tl Allchar mine (Kožuf Mountain, North Macedonia), at the Crven Dol locality, during the full flowering period on 21 June 2024. The geographical coordinates of each sample are given in Table 2. Only material free of adhering soil and dust was taken. The plants were collected and taxonomically identified by K. Bačeva Andonovska (Research Centre for Environment and Materials, Macedonian Academy of Sciences and Arts, Skopje). Voucher specimens of all six taxa are deposited in the Macedonian National Herbarium (MKNH) at the Institute of Biology, Faculty of Natural Sciences and Mathematics, Ss. Cyril and Methodius University in Skopje, which is listed in Index Herbariorum under the acronym MKNH; the registration number of each voucher specimen is given in Table 2.

**TABLE 2:** Collection data for analyzed samples.

Plant family	Plant species	Codes	Date of collection	Locations	Coordinates	Voucher nos.
Violaceae	*Viola arsenica* Beck	VAr	21 June 2024	Crven Dol	41.160; 21.951	242161 (MKNH)
Violaceae	*Viola allchariensis* Beck	VAl	21 June 2024	Crven Dol	41.162; 21.947	242162 (MKNH)
Violaceae	*Viola tricolor* subsp. *macedonica* Boiss. & Heldr	VM	21 June 2024	Crven Dol	41.164, 21.945	242163 (MKNH)
Lamiaceae	*Thymus thracicus Velen*. var. *alsarensis*	TT	21 June 2024	Crven Dol	41.159; 21.948	242164 (MKNH)
Asteraceae	*Centaurea leucomalla* Bornm.	CL	21 June 2024	Crven Dol	41.159; 21.952	242165 (MKNH)
Caprifoliaceae	*Knautia caroli‐rechingeri* Micevski	KC	21 June 2024	Crven Dol	41.162; 21.950	242166 (MKNH)

### 4.2 BVOCs Analysis

Plant material was ground using an electric mill. Approximately 50 mg of the powdered sample was weighed into a GC glass vial, using an analytical balance (±0.01 mg); each sample was prepared and analyzed in triplicate. The samples were analyzed using headspace‐gas‐chromatography‐mass‐spectrometry (HS‐GC–MS) method. The analysis was performed using an Agilent 8890N gas chromatograph coupled to a 5977B mass spectrometer in electron impact ionization mode at 70 eV. An Agilent HP‐5MS UI (30 m × 250 µm × 0.25 µm) column was used with helium as mobile phase with flow rate of 1 mL/min. Samples were incubated at 180°C for 20 min, then injected volume of 1 mL (pulsed split mode 1:10) and injection temperature of 250°C. The oven temperature gradient is given below. The oven temperature programme is given in Table 3:

**TABLE 3:** Temperature programme of the oven used for the HS‐GC–MS analysis.

Rate/(°C/min)	Temperature/°C	Hold time/min
2	80	0
5	180	0
20	260	1

Identification of compounds was made by assignment of their mass spectra and comparing them with the NIST spectral libraries (NIST MS Search 2.3). Relative abundances are expressed as the percentage of the total peak area of the identified compounds, without correction for response factors.

### 4.3 Extraction of Non‐Volatile‐Compounds

The powdered material was accurately weighed (∼0.1 g) on an analytical balance (±0.01 mg). Each taxon was represented by one composite sample of aerial parts pooled from several individuals, and all extractions and instrumental analyses were performed in triplicate. The extraction of polyphenolic compounds was conducted using 15 mL natural deep eutectic solvent composed of choline chloride and acetic acid in molar ratio 1:2, with 40% (*m/m*) water content. Ultrasonic assisted extraction was conducted for 40 min, at 50°C. Extracts were filtered (RC 0.45 µm) and diluted 1:1 (*V/V*) prior to instrumental analysis. The procedure was previously optimized by our research group [60].

For HPLC/DAD/ESI‐MS*^n^* analysis Agilent 1100 Series instrument equipped with a diode‐array detector (DAD) and an ion‐trap mass spectrometer (Agilent Technologies, Waldbronn, Germany) was used. A XDB‐C18 Eclipse column (150 × 4.6 mm, 5 µm, Agilent, USA) was used for chromatographic separation. The mobile phase consisted of water with 1% formic acid (solvent A) and methanol (solvent B). Gradient elution was applied as follows: the run started with 20% B, which was maintained until 5 min, then gradually increased to 40% B at 15 min, 70% B at 45 min, and finally 90% B at 55 min, which was held until 60 min. After each run, a 10‐min equilibration period was applied. The flow rate was 0.5 mL min^−^^1^, the injection volume was 10 µL, and the column temperature was maintained at 38°C.

Spectral data from all peaks were accumulated in the 190–600 nm range, and chromatograms were recorded at 280, 300, 330 and 350 nm. MS and MS [2] spectra were acquired in the negative ionization mode across an *m/z* range of 100–1200 with an electrospray ionization (ESI) system. The nebulizing gas was nitrogen at 65 psi with a flow rate of 12 L/min.

Identification of compounds was based on UV and MS spectral characteristics and comparison with reference standards and literature data.

Hydroxycinnamic acid derivatives were quantified as caffeic acid equivalents at 330 nm. Flavones and flavonols were quantified as luteolin and isorhamnetin equivalents at 350 nm. Iridoids were quantified as catalpol equivalents at 330 nm. Calibration curves for all standards were constructed over the concentration range of 1–500 µmol L^−1^.

### 4.4 Statistical Analysis

Principal component analysis (PCA) was performed on log(1 + *x*)‐transformed and Pareto‐scaled metabolite data to assess patterns in polyphenolic and BVOC composition. Hierarchical clustering was based on Bray–Curtis dissimilarities with average linkage, and cluster stability was evaluated by bootstrap resampling of compounds (2000 replicates). Concordance between polyphenolic and BVOC profiles was assessed using a Mantel test based on Spearman correlation of the corresponding dissimilarity matrices. Differences related to previously reported metal‐handling behavior were evaluated by PERMANOVA. Exploratory Spearman correlations were used to assess relationships between metabolite classes and previously reported aerial‐part Tl concentrations for the five taxa with available data, with Benjamini–Hochberg correction for multiple testing. Due to the limited number of taxa and available published metal data, As and Sb were not included in the correlation analysis. All permutation‐based tests were evaluated by complete enumeration to obtain exact *p*‐values. Statistical analyses were performed using Python 3.11 (NumPy 2.4, SciPy 1.17, scikit‐learn 1.8).

## Author Contributions

M.C. and J.P.S. designed the study. K.B.A. collected and verified the plant samples. M.C. performed the experimental procedures. M.C. and J.P.S. performed the data analysis. M.C. and J.P.S. wrote the manuscript. M.S. reviewed the manuscript. All authors read and approved the final manuscript. All figures, schemes, and tables in this manuscript and in the Supporting Information were created originally by the authors for this work. No material has been reproduced or adapted from previously published sources, and no third‐party copyrighted images, logos or trademarks are included; no copyright permissions are therefore required.

## Conflicts of Interest

The authors declare no conflicts of interest.

## Supporting information




**Supporting File 1**: cbdv71761‐sup‐0001‐SuppMat.docx.

## Data Availability

Data available on request from the authors.
