# Supplementary material for: Polyphenolic and Biogenic Volatile Organic Compound Profiles of Endemic Metallophytes From the Thallium‐Rich Allchar Mining Area
Source: Chem Biodivers. 2026 Sep 27;23(9):e71761. doi: 10.1002/cbdv.71761 (PMC13616439; doi:10.1002/cbdv.71761)
Supplement: Supplementary file 1 [file CBDV-23-e71761-s001.docx]

**Figure S1a:** Representative chromatogram obtained by GC-MS analysis of *Viola macedonica*.

**Figure S1b:** Representative chromatogram obtained by GC-MS analysis of *Viola arsenica*.

**Figure S1c:** Representative chromatogram obtained by GC-MS analysis of *Viola allchariensis*.

**Figure S1d:** Representative chromatogram obtained by GC-MS analysis of *Knautia caroli-rechingeri*.

**Figure S1e:** Representative chromatogram obtained by GC-MS analysis of *Centaurea leucomalla*.

**Figure S1f:** Representative chromatogram obtained by GC-MS analysis of *Thymus thracicus* var. *alsarensis*

**Figure S2:** Representative chromatograms obtained by HPLC-DAD-MS analysis of all plant samples.

**Table S1:** Relative abundance (%) of BVOCs in the analyzed samples obtained by HS-GC-MS (VM – *V. macedonica*, VAl – *V. allchariensis*, VAr – *V. arsenica*, CL – *C. leucomalla*, TA – *T. thracicus* var. *alsarensis*, KC – *K. caroli-rechingeri*)

| **No.** | ***t_R_*/min** | **Compound name** | **VAl** | **VM** | **VAr** | **CL** | **KC** | **TA** |
| --- | --- | --- | --- | --- | --- | --- | --- | --- |
|  | **Aliphatic hydrocarbons (alkanes, alkenes, non-terpene)** | | | | | | | |
| **3** | 1.2 | Heptane | 9.21 | 12.56 | 2.33 | 20.08 | 10.78 | 0.31 |
| **112** | 31.9 | Hexadecane | 0.24 | 0.29 | 0.21 | 0.39 | 0.20 | - |
| **114** | 34.2 | Nonadecane | - | 0.35 | 0.17 | 0.33 | - | - |
| **120** | 37.2 | Eicosane | 1.23 | 2.11 | 0.17 | 0.26 | - | - |
|  |  | **Total** | **10.68** | **15.31** | **2.88** | **21.06** | **10.98** | **0.31** |
|  |  |  |  |  |  |  |  |  |
|  | **Carboxylic acids (aliphatic and aromatic)** | | | | | | | |
| **1** | 0.9 | Acetic acid | 7.27 | 10.29 | 12.03 | 3.19 | 5.95 | - |
| **5** | 1.9 | 2-methylpropanoic acid | 0.24 | 0.83 | - | 0.83 | 0.67 | - |
| **10** | 3.6 | 3-methylbutanoic acid | 0.27 | 0.33 | - | 0.81 | - | - |
| **23** | 8.9 | Propylpropanedioic acid | - | - | - | 0.36 | - | - |
| **24** | 8.9 | Hexanoic acid | - | - | - | - | 0.73 | - |
| **71** | 22.9 | Nonanoic acid | - | - | - | - | 0.51 | - |
|  |  | **Total** | **7.79** | **11.46** | **12.03** | **5.19** | **7.86** | **-** |
|  |  |  |  |  |  |  |  |  |
|  | **Aldehydes (aliphatic and aromatic)** | | | | | | | |
| **2** | 1.0 | 2-methylbutanal | 21.37 | 22.11 | 16.37 | 5.01 | 19.43 | - |
| **9** | 2.7 | Hexanal | 0.37 | 1.00 | 5.40 | 4.33 | - | - |
| **11** | 3.9 | 2-Hexenal | - | - | - | 6.34 | 8.34 | - |
| **13** | 5.4 | Heptanal | 0.50 | 0.33 | 0.17 | 2.17 | - | 0.02 |
| **19** | 7.8 | Benzaldehyde | 1.05 | 1.81 | 1.51 | 1.80 | 1.47 | - |
| **36** | 12.1 | Benzeneacetaldehyde | 5.48 | 6.18 | 4.98 | 10.39 | 10.56 | 0.02 |
| **48** | 15.9 | Nonanal | 1.07 | 1.74 | 1.72 | 7.14 | 1.48 | 0.14 |
| **50** | 16.2 | 2-methyl-3-methylene-cyclopentanecarboxaldehyde | - | - | - | 0.96 | - | - |
| **65** | 20.5 | Decanal | 0.27 | - | - | 5.82 | 0.32 | - |
| **72** | 22.9 | β-methylcinnamaldehyde | 0.24 | 0.40 | 0.48 | - | - | - |
| **115** | 34.5 | Octadecanal | - | - | - | 0.32 | - | - |
|  |  | **Total** | **30.36** | **33.58** | **30.64** | **44.28** | **41.60** | **0.17** |
|  |  |  |  |  |  |  |  |  |
|  | **Alcohols (non-terpene)** | | | | | | | |
| **4** | 1.9 | Propylene glycol | - | - | - | - | 1.35 | - |
| **7** | 2.5 | 2,3-Butanediol | - | - | - | - | 4.70 | - |
| **8** | 2.6 | 3-methyl-2-pentanol | - | - | - | - | 7.71 | - |
| **21** | 8.8 | 1-octen-3-ol | - | - | - | - | - | 0.62 |
| **22** | 8.8 | 2-propyl-tetrahydropyran-3-ol | 0.51 | 0.39 | 0.44 | - | - | - |
| **33** | 11.7 | Benzyl alchohol | - | 0.81 | 0.95 | - | 1.15 | - |
| **44** | 14.4 | 1-Nonen-3-ol | - | - | - | - | - | 0.10 |
| **51** | 16.3 | Phenylethyl alchohol | - | - | - | - | 1.68 | - |
|  |  | **Total** | **0.51** | **1.20** | **1.39** | **-** | **16.59** | **0.72** |
|  |  |  |  |  |  |  |  |  |
|  | **Ketones (non-terpene)** | | | | | | | |
| **12** | 4.9 | 3-Hexanone, 5-methyl | - | - | - | - | - | 0.02 |
| **25** | 9.2 | 3-Octanone | - | - | - | - | - | 0.13 |
| **102** | 30.0 | cis-2-methyl-3-oxo-cyclohexanebutanal | - | 0.06 | 0.28 | 0.47 | 0.28 | - |
| **118** | 36.7 | 6,10,14-trimethyl-2-pentadecanone | 0.59 | 0.74 | 0.25 | 1.04 | - | - |
|  |  | **Total** | **0.59** | **0.80** | **0.53** | **1.51** | **0.28** | **0.15** |
|  |  |  |  |  |  |  |  |  |
|  | **Esters (non-terpene)** | | | | | | | |
| **6** | 2.2 | Methyl isovalerate | - | - | - | - | - | 0.13 |
| **121** | 37.5 | 13-methylpentadecanoic methyl ester | - | - | 0.80 | 0.25 | - | - |
|  |  | **Total** | **-** | **-** | **0.80** | **0.25** | **-** | **0.13** |
|  |  |  |  |  |  |  |  |  |
|  | **Lactones and Furanones** | | | | | | | |
| **15** | 6.0 | Butyrolactone | 2.68 | 2.36 | 4.42 | 0.26 | 2.05 | - |
| **35** | 12.0 | 5-Ethyl-2(5H)-Furanone | - | - | - | 0.37 | 0.51 | - |
| **39** | 13.2 | Furaneol | 0.79 | 0.88 | 2.53 | - | 0.03 | - |
| **57** | 17.7 | DDMP | 22.95 | 5.82 | 9.76 | 0.59 | 5.73 | - |
| **105** | 30.3 | Dihydroactinolide | 0.22 | 1.19 | 0.31 | 1.51 | 0.87 | - |
|  |  | **Total** | **26.64** | **10.25** | **17.02** | **2.73** | **9.19** | **-** |
|  |  |  |  |  |  |  |  |  |
|  | **Nitrogen containing heterocycles** | | | | | | | |
| **14** | 5.8 | 2,5-dimethylpyrazine/2,6-dimethylpirazine | 5.11 | 6.87 | 6.20 | 0.29 | 1.36 | 0.01 |
| **40** | 13.3 | 1-(1H-pyrrol-2-yl)ethanone | 2.56 | 3.24 | 2.08 | - | 2.35 | - |
| **43** | 14.4 | 3-ethyl-2,5-dimethylpyrazine | 1.43 | 2.25 | 1.42 | - | - | - |
| **49** | 16.0 | 6-Azabicyclo[3.2.1]octane | 7.73 | 2.78 | 10.98 | - | - | - |
|  |  | **Total** | **16.83** | **15.14** | **20.68** | **0.29** | **3.71** | **0.01** |
|  |  |  |  |  |  |  |  |  |
|  | **Aromatic hydrocarbons and derivatives** | | | | | | | |
| **68** | 21.1 | 2,3-dihydrobenzofuran | 0.47 | - | 1.29 | - | - | - |
| **109** | 30.9 | (E)-1-(6,10-dimethylundec-5-en-2-yl)-4-methylbenzene | - | - | - | - | - | 0.42 |
|  |  | **Total** | **0.47** | **-** | **1.29** | **-** | **-** | **0.42** |
|  |  |  |  |  |  |  |  |  |
|  | **Phenols and phenolic derivatives** | | | | | | | |
| **74** | 24.2 | Carvacrol (2-methyl-5-(1-methylethyl)phenol) | - | - | - | - | - | 16.24 |
| **75** | 24.3 | 2-Metoxy-4-vinylphenol | - | - | - | - | 0.30 | - |
| **77** | 26.1 | 2-methyl-5-(1-methylethyl)-Phenol acetate | - | - | - | - | - | 0.56 |
|  |  | Total | **-** | **-** | **-** | **-** | **0.30** | **16.80** |
|  |  |  |  |  |  |  |  |  |
|  | **Monoterpene hydrocarbons (C_10_H_16_)** | | | | | | | |
| **17** | 6.7 | α-Pinene/1,3,3-trimethyl-tricyclo[2.2.1.0(2,6)]heptane | - | - | - | - | - | 2.03 |
| **18** | 7.3 | Camphene | - | - | - | - | - | 2.16 |
| **26** | 9.4 | β-Myrcene | - | - | - | - | - | 1.92 |
| **27** | 10.0 | α-Phellandrene | - | - | - | - | - | 0.81 |
| **30** | 11.1 | o-Cymene/p-Cymene/m-Cymene | 0.41 | 0.51 | 0.49 | 0.70 | 0.78 | 6.51 |
| **31** | 11.3 | D-Limonene | - | - | - | - | - | 29.74 |
| **34** | 11.9 | trans-β-Ocimene | - | - | - | - | - | 0.18 |
| **38** | 13.1 | γ-Terpinene | - | - | - | - | - | 7.76 |
| **37** | 13.0 | 3-Carene | - | - | - | 0.55 | 0.54 | 0.10 |
| **46** | 15.4 | 2-Carene | - | - | - | - | - | 0.03 |
| **56** | 17.6 | 4-Carene | - | - | - | - | - | 0.04 |
|  |  | **Total** | **0.41** | **0.51** | **0.49** | **1.25** | **1.32** | **51.28** |
|  |  |  |  |  |  |  |  |  |
|  | **Oxygenated monoterpenes** | | | | | | | |
|  | *Alcohols* | | | | | | | |
| **42** | 13.9 | Isopulegol | - | - | - | - | - | 0.02 |
| **47** | 15.6 | Linalool | - | - | - | - | 0.93 | 0.17 |
| **55** | 17.3 | cis-p-Mentha-2,8-dien-1-ol/Carveol | - | - | - | - | - | 0.03 |
| **59** | 18.8 | endo-Bomeol | - | - | - | - | - | 3.34 |
| **60** | 19.3 | Terpinen-4-ol | - | - | - | - | - | 0.61 |
| **61** | 19.9 | α-Terpineol | - | - | - | - | - | 0.22 |
| **63** | 20.1 | (-)-Myrtenol | - | - | - | - | 1.79 | - |
| **67** | 21.1 | trans-chrysantenol/cis-Crysantenol/2,2,6-trimethyl-1-cyclohexene-1-carboxaldehyde | - | - | - | - | 0.84 | - |
| **73** | 23.9 | Thymol | 2.78 | 0.27 | 0.23 | 19.30 | 4.22 | 0.71 |
| **86** | 28.0 | p-Cymene-2,5-diol | - | - | - | - | - | 0.20 |
|  | *Ketones, aldehydes and oxides* | |  |  |  |  |  |  |
| **32** | 11.4 | Eucalyptol | - | - | - | - | - | 0.08 |
| **53** | 16.9 | α-Campholenal | - | - | - | - | - | 0.02 |
| **58** | 17.4 | Camphor | - | - | - | - | - | 0.03 |
| **64** | 20.2 | D-Verbenone | - | 0.24 | 0.27 | 0.40 | - | - |
| **69** | 21.9 | Carvone | - | - | - | - | - | 0.20 |
| **70** | 22.1 | Thymoquinone | - | - | - | - | - | 2.35 |
|  | *Esters* | | | | | | | |
| **41** | 13.5 | Piperityl acetate | - | - | - | - | - | 0.47 |
| **52** | 16.7 | 4-Terpenyl acetate | - | - | - | - | - | 0.10 |
|  |  | **Total** | **2.78** | **0.51** | **0.51** | **19.69** | **7.78** | **8.54** |
|  |  |  |  |  |  |  |  |  |
|  | **Sesquiterpene hydrocarbons (C_15_H_24_)** | | | | | | | |
| **76** | 25.9 | α-Copaene | - | - | - | - | - | 0.09 |
| **78** | 26.2 | Cubenene | - | - | - | - | - | 0.13 |
| **79** | 26.4 | β-Bourbonene | - | - | - | - | - | 0.26 |
| **80** | 26.6 | cis-Muurola-4(15), 5-diene | - | - | - | - | - | 0.07 |
| **81** | 26.6 | α-Guainene | - | - | - | - | - | 0.04 |
| **82** | 27.4 | Caryophyllene | - | - | 0.50 | 1.51 | - | 1.89 |
| **83** | 27.7 | β-Copaene | - | - | - | - | - | 0.19 |
| **84** | 27.8 | trans-α-Bergamotene/cis-α-Bergamotene | - | - | - | - | - | 0.09 |
| **85** | 27.9 | Aromadendrene | - | - | - | - | - | 0.09 |
| **88** | 28.3 | Humulene | - | - | - | - | - | 0.08 |
| **89** | 28.4 | β-Farnesene | - | - | - | - | - | 0.06 |
| **90** | 28.9 | γ-Muurolene | - | - | - | - | - | 0.31 |
| **91** | 29.1 | Gemacrene D | - | - | - | - | - | 0.85 |
| **93** | 29.1 | Cedrene | - | - | - | - | - | 0.07 |
| **96** | 29.4 | Ylangene | - | - | - | - | - | 0.20 |
| **97** | 29.5 | γ-Elemene | - | - | - | - | - | 0.21 |
| **98** | 29.5 | α-Muurolene | - | - | - | - | - | 0.16 |
| **106** | 30.4 | Cubenene | - | - | - | - | - | 0.05 |
| **100** | 29.8 | β-Bisabolene | - | - | - | - | - | 3.34 |
| **111** | 31.6 | β-Himachlene/γ-Himachlene | - | - | - | 1.19 | - | 0.04 |
|  |  | **Total** | **-** | **-** | **0.50** | **2.69** | **-** | **8.20** |
|  |  |  |  |  |  |  |  |  |
|  | **Oxygenated sesquiterpenes** | | | | | | | |
| **99** | 29.8 | trans-Sesquisabinene hydrate | - | - | - | 1.64 | 0.20 | - |
| **110** | 31.5 | (-)-Spathulenol | - | - | - | - | - | 0.18 |
|  |  | **Total** | **-** | **-** | **-** | **1.64** | **0.20** | **0.18** |
|  |  |  |  |  |  |  |  |  |
|  | **Higher terpenoids/ Polycyclic terpenes** | | | | | | | |
| **94** | 29.2 | trans-β-Ionone | 0.35 | 1.55 | 0.57 | 0.88 | 1.27 | - |
| **16** | 6.4 | Alpha-Thujene | - | - | - | - | - | 2.48 |
| **20** | 8.6 | β-Terpinene | - | - | - | - | - | 0.76 |
| **28** | 10.6 | 7-norbornenol | - | - | - | 1.28 | 0.31 | - |
| **29** | 10.7 | α-Terpinene | - | - | - | - | - | 2.94 |
| **45** | 14.8 | Terpinolene | - | - | - | - | - | 0.65 |
| **54** | 17.1 | (4E,6Z)-allo-Ocimene | - | - | - | - | - | 0.02 |
| **62** | 20.0 | Methyl salicilate | 2.09 | 7.65 | 9.34 | - | - | 0.09 |
| **66** | 21.0 | trans-Chrysantenol/cis-Chrysanthenol | - | 0.63 | - | 0.65 | - | - |
| **87** | 28.2 | cadina-1(6),4-diene | - | - | - | - | - | 0.05 |
| **92** | 29.1 | dehydro-β-ionone | 0.22 | 0.69 | 0.66 | - | - | - |
| **95** | 29.3 | 3-ethyl-4,4-dimethyl-2-(2-methylpropenil)cyclohex-2-enone | - | - | 0.22 | - | - | - |
| **101** | 29.9 | γ-Cadinene | - | - | - | - | - | 0.24 |
| **103** | 30.1 | δ-cadinene | - | - | - | - | - | 0.57 |
| **104** | 30.2 | cis-1-methylbicyclodecane-2,10-dione | 0.30 | 0.74 | 0.45 | 0.93 | 0.66 | - |
| **107** | 30.5 | (-)-α-Cadinene | - | - | - | - | - | 0.05 |
| **108** | 30.6 | trans-α-bisabolene | - | - | - | - | - | 0.08 |
| **113** | 33.4 | ent-13-epi-manoyl oxide | - | - | - | - | - | 1.74 |
| **116** | 34.7 | Palustradiene | - | - | - | - | - | 1.65 |
| **117** | 36.2 | Dehydroabietane (stereoisomer) | - | - | - | - | - | 0.83 |
| **119** | 36.7 | (4aS, 4bR, 10aS)-7-Isopropyl-1,1,4a-trimethyl-1,2,3,4,4a,4b,5,6,10,10a-decahydrophenantrene | - | - | - | - | - | 0.10 |
| **122** | 38.6 | Dehydroabietane (stereoisomer) | - | - | - | - | - | 0.04 |
| **123** | 38.9 | 3β-Hydroxymanool (stereoisomer) | - | - | - | - | - | 0.80 |
|  |  | **Total** | **2.95** | **11.25** | **11.25** | **3.73** | **2.23** | **13.10** |
|  |  |  |  |  |  |  |  |  |

**Table S2:** Content of individual compounds detected in analyzed samples by HPLC-DAD-MS, expressed in mg/g dry herb, analyzed in triplicates (VM – *V. macedonica*, VAl – *V. allchariensis*, VAr – *V. arsenica*, CL – *C. leucomalla*, TA – *T. thracicus* var*. alsarensis*, KC – *K. caroli-rechingeri*)

| **No.** | ***t_R_*/min** | **Compound name** | **VM** | | **VAl** | **VAr** | | **CL** | | | **TA** | **KC** | |
| --- | --- | --- | --- | --- | --- | --- | --- | --- | --- | --- | --- | --- | --- |
| **Phenolic acids** | | | | | | | | | | | | | |
| **Caffeoyl derivatives** | | | | | | | | | | | | | |
|  | *Mono- and dicaffeoyl derivatives* | |  | |  |  | |  | | |  |  | |
| **2** | 7.5 | 3-Caffeoylquinic acid | - | | - | - | | 1.01 | | | - | 0.53 | |
| **7** | 13.3 | 5-Caffeoylquinic acid | - | | - | - | | 17.99 | | | - | 28.93 | |
| **10** | 15.4 | Caffeic acid | - | | - | - | | - | | | 0.16 | - | |
| **12** | 16.6 | 4-Caffeoylquinic acid | - | | - | - | | - | | | - | 0.87 | |
| **35** | 22.2 | 1,3-Dicaffeoylquinic acid | - | | - | - | | - | | | - | - | |
| **50** | 25.1 | 1,4-Dicaffeoylquinic acid | - | | - | - | | - | | | - | 2.12 | |
| **54** | 26.7 | 3,4-Dicaffeoylquinic acid | - | | - | - | | 4.81 | | | - | - | |
| **56** | 27.5 | 1,5-Dicaffeoylquinic acid | - | | - | - | | 4.50 | | | - | - | |
| **62** | 29.9 | 3,5-Dicaffeoylquinic acid | - | | - | - | | - | | | - | 0.68 | |
| **64** | 30.4 | 4,5-Dicaffeoylquinic acid | - | | - | - | | 0.79 | | | - | - | |
|  | *Salvianolic acids* | |  | |  |  | |  | | |  |  | |
| **46** | 24.5 | Salvianolic acid A | - | | - | - | | - | | | 0.16 | - | |
| **53** | 26.6 | Salvianolic acid K | - | | - | - | | - | | | 2.06 | - | |
| **73** | 34.3 | Isosalvianolic acid A | - | | - | - | | - | | | 2.00 | - | |
| **75** | 36.8 | Salvianolic Acid F | - | | - | - | | - | | | 0.07 | - | |
|  | *Glycosides* |  | - | | - | - | | - | | | - | - | |
| **4** | 9.6 | Caffeic acid 3-*O*-glucoside | - | | - | - | | - | | | - | 0.06 | |
|  | *Depsides (caffeic acid esters)* | | | | | | | | | | | | |
| **33** | 21.3 | Rosmarinic acid glycoside | - | | - | - | | - | | | 0.08 | - | |
| **47** | 24.9 | Rosmarinic acid isomer | - | | - | - | | - | | | 0.05 | - | |
| **52** | 25.9 | Rosmarinic acid | - | | - | - | | - | | | 5.76 | - | |
| **Hydroxybenzoic acids derivatives** | | | | | | | | | | | | | |
| **1** | 6.7 | Gentisoyl glucoside (Gentisic acid glucoside) | - | - | | | 0.66 | | - | - | | | - |
| **3** | 7.8 | Protocatechuic Acid Glucoside | - | | - | 0.17 | | - | | | - | - | |
| **5** | 10.2 | Protocatechuic Acid Glucoside + Pentose | - | | - | 0.05 | | - | | | - | - | |
| **6** | 13.1 | Hydroxybenzoic acid Glucoside | 0.21 | | 0.16 | 0.12 | | - | | | - | - | |
| **Coumaroyl derivatives** | | | | | | | | | | | | | |
| **13** | 16.9 | Coumaroyl pentoside | - | | - | - | |  | | | - | 0.75 | |
| **21** | 19.3 | Coumaroylquinic acid | - | | - | - | | 0.21 | | | - | 0.40 | |
| **Feruloyl derivatives** | | | | | | | | | | | | | |
| **19** | 18.3 | Feruoyl arabinoside | - | | - | - | | - | | | - | 1.09 | |
| **32** | 20.9 | 5-Feruoylquinic acid | - | | - | - | | 1.39 | | | - | - | |
| **51** | 25.9 | Caffeoyl feruloylquinic acid | - | | - | - | |  | | | - | 0.16 | |
| **65** | 30.7 | Feruloyl tartaric acid (fertaric acid) | - | | - | - | | - | | | - | 0.11 | |
| **Other** | | | | | | | | | | | | | |
| **8** | 14.1 | Sinapic acid hexoside | - | | - | 0.08 | | - | | | - | - | |
| **14** | 16.9 | Dimer of 3-(3,4-dihydroxyphenyl) lactic acid | - | | - | - | | - | | | 0.49 | - | |
| **16** | 17.9 | Hydroxy jasmonic acid *O*-hexoside | - | | - | - | | - | | | 0.03 | - | |
|  |  | **Total** | **0.21** | | **0.16** | **0.41** | | **31.37** | | | **10.85** | **441.11** | |
|  |  |  |  | |  |  | |  | | |  |  | |
| **Flavan-3-ols** | | | | | | | | | | | | | |
| **11** | 15.9 | Catechin | 0.09 | | - | 0.70 | | - | | | - | - | |
|  |  | **Total** | **0.09** | | - | **0.70** | | - | | | - | - | |
|  |  |  |  | |  |  | |  | | |  |  | |
| **Flavonols** | | | | | | | | | | | | | |
| **Quercetin derivatives** | | | | | | | | | | | | | |
| **15** | 16.9 | Quercetin di-*C*-hexoside | - | | - | - | | 0.26 | | | - | - | |
| **18** | 18.1 | Quercetin di-*C*-hexoside | - | | - | - | | 0.30 | | | - | - | |
| **29** | 20.7 | Quercetin-7-*O*-glucoside | - | | - | - | | - | | | 2.50 | - | |
| **30** | 20.7 | Quercetin-rhamnosyl-rutinoside | - | | 2.50 | - | | - | | | - | - | |
| **48** | 24.9 | Isorhamnetin-rhamnosyl-rutinoside | - | | - | 0.73 | | - | | | - | - | |
| **49** | 24.9 | Quercetin-3-*О*-hexoside-7-*О*-pentoside | - | | - | - | | 0.57 | | | - | - | |
| **58** | 28.5 | Isorhamnetin 3-*O*-hexoside-7-*O*-rhamnoside | 0.25 | | 0.12 | 1.55 | | - | | | - | - | |
| **59** | 28.7 | Patuletin 3-*O*-hexoside | - | | - | - | | 0.23 | | | - | - | |
| **63** | 30.2 | Isorhamnetin (3-*O*-methylquercetin) | - | | - | - | | - | | | 0.15 | - | |
| **67** | 31.1 | Quercetin-*O*-acetylhexoside | - | | - | - | | 0.75 | | | - | - | |
| **70** | 31.8 | Patuletin acetyl hexoside | - | | - | - | | 1.03 | | | - | - | |
| **Kaempferol derivatives** | | | | | | | | | | | | | |
| **23** | 19.4 | Kampferol-*O*-deoxyhexoside-*O*-feruloylrutinoside | - | | - | 0.63 | | - | | | - | - | |
| **28** | 20.5 | Kaempferol 3-*O*-glucoside | 5.58 | |  | 0.89 | | - | | | - | - | |
| **57** | 27.9 | Kaempferol-3-*O*-rutinoside | 0.33 | | 0.11 | 0.60 | | - | | | - | - | |
| **60** | 28.9 | Kaemferol dipentoside | - | | - | - | | - | | | 0.27 | - | |
| **66** | 30.8 | Kaemferol acetyl pentosyl-hexoside | - | | - | - | | - | | | 0.15 | - | |
| **72** | 32.9 | Kaempferol-*O*-acetylhexoside | - | | - | - | | 0.07 | | | - | - | |
| **76** | 38.2 | Kaempferol 3-(6''-acetylglucoside)-7-rhamnoside | - | | - | - | | - | | | - | 0.14 | |
| **Other** | | | | | | | | | | | | | |
| **36** | 22.2 | Myricetin-hexoside | - | | - | - | | 0.99 | | | - | - | |
| **74** | 34.7 | Flavanomarein | - | | - | - | | - | | | - | 0.04 | |
|  |  | **Total** | **6.16** | | **2.73** | **4.40** | | **4.20** | | | **3.08** | **0.19** | |
|  |  |  |  | |  |  | |  | | |  |  | |
| **Flavones** | | | | | | | | | | | | | |
| **Apigenin derivatives** | | | | | | | | | | | | | |
| **17** | 18.0 | Vicenin-2 (Apigenin-6,8-di-*C*-glucoside) | 2.16 | | 0.48 | 1.46 | | - | | | - | - | |
| **22** | 19.3 | Apigenin 6-*C*-glucosyl-7-*O*-glucoside | - | | - | - | | - | | | - | 0.99 | |
| **24** | 19.6 | Apigenin 6-*C*-glucoside 8-*C*-arabinoside (Schaftoside) | 0.24 | | 0.10 | - | | - | | | - | - | |
| **27** | 20.5 | Apigenin-di-*C*-glucoside | - | | - | - | | 1.76 | | | - | - | |
| **37** | 22.3 | Apigenin-*C*-rhamnoside-*C*-hexoside isomer | 2.75 | | 1.24 | 0.76 | | - | | | - | - | |
| **38** | 22.6 | Apigenin-8-*C*-pentoside-6-*C*-hexoside | - | | - | - | | 0.39 | | | - | - | |
| **40** | 23.0 | Apigenin-*C*-rhamnoside-*C*-hexoside isomer | - | | - | 0.80 | | - | | | - | - | |
| **42** | 23.3 | Apigenin-8-*C*-hexoside-6-*C*-pentoside | - | | - | - | | 0.39 | | | - | - | |
| **43** | 23.4 | Apigenin-*C*-rhamnoside-*C*-hexoside isomer | 9.20 | | - | 3.56 | | - | | | - | - | |
| **44** | 23.8 | Acacetin *C*-glucoside | - | | - | - | | - | | | - | 2.78 | |
| **45** | 24.1 | Apigenin-6-*C*-glucoside-8-*C*-rhamnoside | 25.62 | | 12.66 | 15.74 | | - | | | - | - | |
| **Luteolin derivatives** | | | | | | | | | | | | | |
| **25** | 20.0 | luteolin 6-*C*-hexoside-8-*C*-pentoside | - | | - | 0.22 | | - | | | - |  | |
| **26** | 20.4 | Isoorientin (Luteolin-6-*C*-glucoside) | - | | - | - | | - | | | - | 1.17 | |
| **31** | 20.9 | Chrysoeriol *C*-glucoside | - | | - | - | | - | | | - | 6.03 | |
| **34** | 21.6 | Luteolin-8-*C*-(6”-acetyl)-*β*-*D*-glucopyranosid | 0.30 | | - | - | | - | | | - | - | |
| **41** | 23.3 | Luteolin-7-*O*-glucoside | - | | - | - | | - | | | 5.18 | - | |
| **55** | 27.4 | Luteolin-4’-*O*-glucoside | - | | - | - | | - | | | - | 0.31 | |
| **61** | 29.8 | Luteolin hexoside | - | | - | - | | - | | | 0.13 | - | |
| **68** | 31.3 | Luteolin dihexoside | - | | - | - | | - | | | 0.23 | - | |
| **71** | 32.4 | Luteolin acetyl dipentoside | - | | - | - | | - | | | 0.47 | - | |
| **Isoflavone** | | | | | | | | | | | | | |
| **39** | 22.7 | Genistein-*C*-rhamnoside-*C*-hexoside | - | | 0.28 | - | | - | | | - | - | |
|  |  | **Total** | **40.27** | | **14.76** | **22.54** | | **2.54** | | | **6.01** | **11.26** | |
|  |  |  |  | |  |  | |  | | |  |  | |
| **Iridoids and Secoiridoids** | | | | | | | | | | | | | |
| **9** | 15.4 | Oleoside or Secologanoside, secoiridoid | - | | - | - | | - | | | - | 0.68 | |
| **20** | 18.9 | Loganic acid glucoside, iridoid | 0.42 | | 0.27 | - | | - | | | - | - | |
| **77** | 41.8 | p-Coumaroylgeniposidic acid | - | | - | - | | - | | | 0.08 | - | |
| **69** | 31.5 | Unknown 1 | 0.13 | | 0.11 | 0.18 | | - | | |  | - | |
|  |  | **Total** | **0.55** | | **0.38** | **0.18** | | - | | | **0.08** | **0.68** | |
|  |  |  |  | |  |  | |  | | |  |  | |
| **Fatty acids** | | | | | | | | | | | | | |
| **78** | 46.7 | Trihydroxy-octadecenoic acid | - | | - | - | | - | | | 0.84 | - | |
|  |  | **Total** | - | | - | - | | - | | | **0.84** | - | |
|  |  |  |  | |  |  | |  | | |  |  | |
|  |  | **Total all** | **47.28** | | **18.04** | **28.23** | | **38.12** | | | **20.86** | **453.23** | |

**Table S3:** Concentrations of Tl, As and Sb reported in the literature for aerial parts of the six taxa analysed in this work, used for the rank-correlation analysis in Figure 5F. The values were not measured in the present material; they come from earlier studies on plants collected at the same locality but not on the same individuals and not in the same season, and they refer in part to different organs. n.r. = not reported for this taxon.

| **Code** | **Taxon** | **Organ** | **Tl /  mg kg^-1^** | **As /  mg kg^-1^** | **Sb /  mg kg^-1^** | **Metal-handling behaviour** | **Source*** |
| --- | --- | --- | --- | --- | --- | --- | --- |
| VM | *Viola tricolor* subsp. *macedonica* | leaves | 4292 | n.r. | n.r. | Tl hyperaccumulator | [6, 23, 58] |
| VAl | *Viola allchariensis* | leaves | 4013 | n.r. | n.r. | Tl hyperaccumulator | [6, 23, 58] |
| VAr | *Viola arsenica* | leaves | 10400 | < 70 (shoots, leaves, flowers) | n.r. | Tl hyperaccumulator | [6, 23, 58] |
| CL | *Centaurea leucomalla* | aerial parts | n.r. | n.r. | n.r. | excluder (metals retained in roots) | [53] |
| TA | *Thymus thracicus* var. *alsarensis* | leaves / flowers | 104 / 131 | 0.5-102 (leaves) | n.r. | partitioner (Tl to shoots, As retained in roots) | [6], [26], [55] |
| KC | *Knautia caroli-rechingeri* | leaf / stem | 10.4 / 26.3 | 3.78 / 1.66 | < 0.50 | excluder (metals retained in roots) | [25] |
| *Reference numbers refer to the reference list of the main manuscript. Maximum Tl and As values of 58 900 and 381 mg kg^-1^ quoted in the main text for Allchar violets [24, 59] are maxima from synchrotron and survey work and were not used in the correlation analysis. Antimony has been determined in only one of the six taxa and always below the limit of quantification, so no correlation could be computed for this element; arsenic values are available for three taxa only and are partly censored ("< 70") or given as a range, and were likewise not used. | | | | | | | |

**Table S4:** Compound-class composition of the six samples, expressed as the percentage of the total content of identified compounds, calculated from Table S2 (polyphenols) and Table S1 (volatiles). These are the variables used for the rank correlations of Figure 5F.

| **Compound class** | **VM** | **VAl** | **VAr** | **CL** | **TA** | **KC** |
| --- | --- | --- | --- | --- | --- | --- |
| **Polyphenol classes (% of total polyphenol content)** | | | | | | |
| Apigenin derivatives | 84.54 | 80.31 | 77.23 | 6.78 | 0.00 | 0.83 |
| Coumaroyl derivatives | 0.00 | 0.00 | 0.00 | 0.56 | 0.00 | 0.25 |
| Depsides (caffeic acid esters) | 0.00 | 0.00 | 0.00 | 0.00 | 28.24 | 0.00 |
| Fatty acids | 0.00 | 0.00 | 0.00 | 0.00 | 4.03 | 0.00 |
| Feruloyl derivatives | 0.00 | 0.00 | 0.00 | 3.71 | 0.00 | 0.30 |
| Flavan-3-ols | 0.19 | 0.00 | 2.42 | 0.00 | 0.00 | 0.00 |
| Hydroxybenzoic acids derivatives | 0.44 | 0.89 | 3.46 | 0.00 | 0.00 | 0.00 |
| Iridoids and Secoiridoids | 1.16 | 2.11 | 0.62 | 0.00 | 0.38 | 0.15 |
| Isoflavone | 0.00 | 1.55 | 0.00 | 0.00 | 0.00 | 0.00 |
| Kaempferol derivatives | 12.50 | 0.61 | 7.34 | 0.19 | 2.01 | 0.03 |
| Luteolin derivatives | 0.63 | 0.00 | 0.76 | 0.00 | 28.81 | 1.66 |
| Mono- and dicaffeoyl derivatives | 0.00 | 0.00 | 0.00 | 77.72 | 0.77 | 96.75 |
| Other | 0.00 | 0.00 | 0.28 | 2.64 | 2.49 | 0.01 |
| Quercetin derivatives | 0.53 | 14.53 | 7.89 | 8.39 | 12.70 | 0.00 |
| Salvianolic acids | 0.00 | 0.00 | 0.00 | 0.00 | 20.57 | 0.01 |
| **Volatile classes (% of total relative abundance)** | | | | | | |
| Alcohols | 0.27 | 2.78 | 0.23 | 18.50 | 5.30 | 7.62 |
| Alcohols (non-terpene) | 1.20 | 0.51 | 1.39 | 0.00 | 0.72 | 16.26 |
| Aldehydes (aliphatic and aromatic) | 33.57 | 30.35 | 30.64 | 42.44 | 0.18 | 40.76 |
| Aliphatic hydrocarbons (alkanes, alkenes, non-terpene) | 15.31 | 10.68 | 2.88 | 20.18 | 0.31 | 10.76 |
| Aromatic hydrocarbons and derivatives | 0.00 | 0.47 | 1.29 | 0.00 | 0.42 | 0.00 |
| Carboxylic acids (aliphatic and aromatic) | 11.45 | 7.78 | 12.03 | 4.97 | 0.00 | 7.70 |
| Esters | 0.00 | 0.00 | 0.00 | 0.00 | 0.57 | 0.00 |
| Esters (non-terpene) | 0.00 | 0.00 | 0.80 | 0.24 | 0.13 | 0.00 |
| Higher terpenoids/ Polycyclic terpenes | 11.26 | 2.96 | 11.24 | 3.58 | 13.08 | 2.20 |
| Ketones (non-terpene) | 0.80 | 0.59 | 0.53 | 1.45 | 0.15 | 0.27 |
| Ketones, aldehydes and oxides | 0.24 | 0.00 | 0.27 | 0.38 | 2.68 | 0.00 |
| Lactones and Furanones | 10.25 | 26.64 | 17.02 | 2.62 | 0.00 | 9.01 |
| Monoterpene hydrocarbons (C_10_H_16_) | 0.51 | 0.41 | 0.49 | 1.20 | 51.26 | 1.29 |
| Nitrogen containing heterocycles | 15.14 | 16.83 | 20.68 | 0.28 | 0.01 | 3.64 |
| Oxygenated sesquiterpenes | 0.00 | 0.00 | 0.00 | 1.57 | 0.18 | 0.20 |
| Phenols and phenolic derivatives | 0.00 | 0.00 | 0.00 | 0.00 | 16.79 | 0.29 |
| Sesquiterpene hydrocarbons (C_15_H_24_) | 0.00 | 0.00 | 0.50 | 2.59 | 8.22 | 0.00 |
| CL – *C. leucomalla*, KC – K. *caroli-rechingeri*, TA – *T. thracicus* var. *alsarensis*, VAl – *V. allcharinesis*,  VAr – *V. arsenica*, VM – *V. macedonica* | | | | | | |

**Table S5:** Spearman rank correlation between the share of each compound class (Table S4) and the aerial-part Tl concentration reported for the same taxa (Table S3, n = 5 taxa with Tl data). *p*-Values were obtained by complete enumeration of all permutations; p(min) is the smallest p value that the test can return for that class given its pattern of ties, and p(BH) is the Benjamini-Hochberg adjusted value computed jointly over the whole family of 32 classes from both data sets. No class remains significant after correction.

| **Compound class** | **Spearman rho** | ***p* (exact)** | ***p* (smallest attainable)** | ***p* (BH-adjusted)** |
| --- | --- | --- | --- | --- |
| **Polyphenol classes** |  |  |  |  |
| Hydroxybenzoic acids derivatives | 0.872 | 0.1000 | 0.0333 | 0.533 |
| Flavan-3-ols | 0.894 | 0.1000 | 0.1000 | 0.533 |
| Mono- and dicaffeoyl derivatives | -0.894 | 0.1000 | 0.1000 | 0.533 |
| Kaempferol derivatives | 0.800 | 0.1333 | 0.0167 | 0.533 |
| Salvianolic acids | -0.783 | 0.2000 | 0.1000 | 0.640 |
| Apigenin derivatives | 0.600 | 0.3500 | 0.0167 | 0.711 |
| Iridoids and Secoiridoids | 0.600 | 0.3500 | 0.0167 | 0.711 |
| Coumaroyl derivatives | -0.707 | 0.4000 | 0.4000 | 0.711 |
| Feruloyl derivatives | -0.707 | 0.4000 | 0.4000 | 0.711 |
| Luteolin derivatives | -0.500 | 0.4500 | 0.0167 | 0.758 |
| Quercetin derivatives | 0.200 | 0.7833 | 0.0167 | 0.985 |
| Fatty acids | -0.354 | 0.8000 | 0.4000 | 0.985 |
| Depsides (caffeic acid esters) | -0.354 | 0.8000 | 0.4000 | 0.985 |
| Other | -0.154 | 0.8333 | 0.0333 | 0.988 |
| Isoflavone | 0.000 | 1.0000 | 0.4000 | 1.000 |
| **Volatile classes** |  |  |  |  |
| Alcohols | -1.000 | 0.0167 | 0.0167 | 0.533 |
| Carboxylic acids (aliphatic and aromatic) | 0.900 | 0.0833 | 0.0167 | 0.533 |
| Oxygenated sesquiterpenes | -0.894 | 0.1000 | 0.1000 | 0.533 |
| Nitrogen containing heterocycles | 0.800 | 0.1333 | 0.0167 | 0.533 |
| Phenols and phenolic derivatives | -0.783 | 0.2000 | 0.1000 | 0.640 |
| Ketones (non-terpene) | 0.600 | 0.3500 | 0.0167 | 0.711 |
| Monoterpene hydrocarbons (C_10_H_16_) | -0.600 | 0.3500 | 0.0167 | 0.711 |
| Lactones and Furanones | 0.600 | 0.3500 | 0.0167 | 0.711 |
| Aromatic hydrocarbons and derivatives | 0.564 | 0.4000 | 0.0333 | 0.711 |
| Esters (non-terpene) | 0.447 | 0.5000 | 0.1000 | 0.800 |
| Ketones, aldehydes and oxides | 0.308 | 0.6000 | 0.0333 | 0.914 |
| Higher terpenoids/ Polycyclic terpenes | 0.300 | 0.6833 | 0.0167 | 0.985 |
| Esters | -0.354 | 0.8000 | 0.4000 | 0.985 |
| Alcohols (non-terpene) | -0.100 | 0.9500 | 0.0167 | 1.000 |
| Aldehydes (aliphatic and aromatic) | -0.100 | 0.9500 | 0.0167 | 1.000 |
| Aliphatic hydrocarbons (alkanes, alkenes, non-terpene) | 0.000 | 1.0000 | 0.0167 | 1.000 |
| Sesquiterpene hydrocarbons (C_15_H_24_) | 0.112 | 1.0000 | 0.1000 | 1.000 |
| For reference, the total polyphenol content gives rho = -0.200 (p = 0.783) and the number of volatile compounds detected  rho = -0.500 (p = 0.450) against the same Tl values. | | | | |

**Table S6:** Summary of the multivariate tests shown in Figure 5. All permutation tests were evaluated by complete enumeration; the number of distinct permutations and the resulting floor on the attainable *p* value are given for each test.

| **Test** | **Data set** | **Statistic** | **p (exact)** | **Permutations** | **p floor** |
| --- | --- | --- | --- | --- | --- |
| Mantel (Spearman) of the two Bray-Curtis matrices | polyphenols vs volatiles | rho = 0.720 | 0.0083 | 720 | 0.0014 |
| Mantel, sensitivity check (Euclidean, Pareto-scaled) | polyphenols vs volatiles | rho = 0.850 | 0.0014 (= floor) | 720 | 0.0014 |
| PERMANOVA, hyperaccumulating vs non-hyperaccumulating | polyphenols | F = 3.67 | 0.100 (= floor) | 20 | 0.100 |
| PERMANOVA, hyperaccumulating vs non-hyperaccumulating | volatiles | F = 2.06 | 0.100 (= floor) | 20 | 0.100 |
| PCA, variance explained by PC1 / PC2 | polyphenols | 37.3 % / 31.9 % | - | - | - |
| PCA, variance explained by PC1 / PC2 | volatiles | 53.5 % / 24.7 % | - | - | - |
| Bootstrap support, *Viola* group (2000 resamplings) | polyphenols | 100 % | - | - | - |
| Bootstrap support, *Viola* group (2000 resamplings) | volatiles | 89 % | - | - | - |
| Bootstrap support, *C. leucomalla* + *K. caroli-rechingeri* | polyphenols | 95 % | - | - | - |
| Bootstrap support, all taxa except *T. thracicus var. alsarensis* | volatiles | 100 % | - | - | - |
